# Overcoming the Limits of Cross-Sensitivity: Pattern Recognition Methods for Chemiresistive Gas Sensor Array

**DOI:** 10.1007/s40820-024-01489-z

**Published:** 2024-08-14

**Authors:** Haixia Mei, Jingyi Peng, Tao Wang, Tingting Zhou, Hongran Zhao, Tong Zhang, Zhi Yang

**Affiliations:** 1 Key Lab Intelligent Rehabil & Barrier Free Disable (Ministry of Education), Changchun University, Changchun, 130022 People’s Republic of China; 2 Shanghai Key Laboratory of Intelligent Sensing and Detection Technology, School of Mechanical and Power Engineering, East China University of Science and Technology, Shanghai, 200237 People’s Republic of China; 3 National Key Laboratory of Advanced Micro and Nano Manufacture Technology, Department of Micro/Nano Electronics, School of Electronic Information and Electrical Engineering, Shanghai Jiao Tong University, Shanghai, 200240 People’s Republic of China; 4 State Key Laboratory of Integrated Optoelectronics, College of Electronic Science and Engineering, Jilin University, Changchun, 130012 People’s Republic of China

**Keywords:** Pattern recognition, Sensor array, Chemiresistive gas sensor, Cross-sensitivity, Artificial olfactory

## Abstract

As information acquisition terminals for artificial olfaction, chemiresistive gas sensors are often troubled by their cross-sensitivity, and reducing their cross-response to ambient gases has always been a difficult and important point in the gas sensing area. Pattern recognition based on sensor array is the most conspicuous way to overcome the cross-sensitivity of gas sensors. It is crucial to choose an appropriate pattern recognition method for enhancing data analysis, reducing errors and improving system reliability, obtaining better classification or gas concentration prediction results. In this review, we analyze the sensing mechanism of cross-sensitivity for chemiresistive gas sensors. We further examine the types, working principles, characteristics, and applicable gas detection range of pattern recognition algorithms utilized in gas-sensing arrays. Additionally, we report, summarize, and evaluate the outstanding and novel advancements in pattern recognition methods for gas identification. At the same time, this work showcases the recent advancements in utilizing these methods for gas identification, particularly within three crucial domains: ensuring food safety, monitoring the environment, and aiding in medical diagnosis. In conclusion, this study anticipates future research prospects by considering the existing landscape and challenges. It is hoped that this work will make a positive contribution towards mitigating cross-sensitivity in gas-sensitive devices and offer valuable insights for algorithm selection in gas recognition applications.

## Highlights

- The types, working principles, advantages and limitations of pattern recognition methods based on chemiresistive gas sensor array are reviewed and discussed comprehensively.
- Outstanding and novel advancements in the application of machine learning methods for gas recognition in different important areas are compared, summarized and evaluated.
- The current challenges and future prospects of machine learning methods in artificial olfactory systems are discussed and justified.

## Graphical abstract

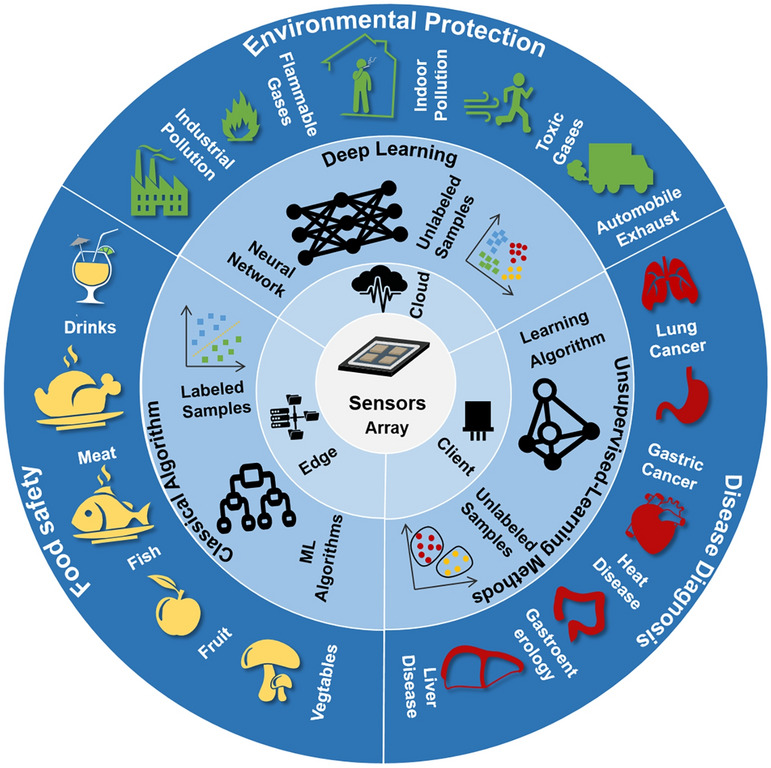

## Introduction

Our olfactory sense plays a crucial role in perceiving and understanding the world around us [1]. Our sense of smell in daily life can assist us in detecting and distinguishing odors in abnormal ranges, tracking odor trajectories, and it can also function as a warning mechanism in dangerous situations [2]. However, the hazardous, caustic, volatile, and combustible gas environment endangers human health, and cannot be effectively detected and analyzed solely by the human sense of smell.

Dedicated researchers are actively working towards resolving these challenges, leading to the development of commercial gas sensors. The first gas sensor was invented in 1968 by Figaro Corporation, named Taguchi Gas Sensor [3]. It is widely used for the detection of hazardous gas leaks in domestic gas and industrial safety. Subsequently, a variety of new sensors have emerged, including electrochemical, catalytic combustion, thermally conductive, infrared absorption gas sensors, and more. Chemiresistive gas sensors have undoubtedly become the most widely used gas sensors due to their high sensitivity, wide range of material sources, simple manufacturing, and cost-effectiveness [4]. Meanwhile, chemiresistive materials with different components [5, 6], morphologies [7] and structures [8] have been continuously developed to improve the performance of gas sensors [9, 10]. They have been widely studied and applied in food quality [11, 12], environmental protection [13, 14] and medical diagnosis [15–17].

In practice, gas sensors typically operate in complex atmospheric environments that contain a large number of interfering gas molecules. Due to the inherent characteristics of the sensitive material, the gas sensor responds not only to the target gas but also to other gases. This manifests as the sensor’s cross-sensitivity to the ambient gas. It can make the detection result inaccurate, and lead to false or missed alarms in practical applications, which greatly limits the practical application of the chemiresistive gas sensor [18]. For example, alcohol is an important interfering gas for various semiconductor gas alarms, and various misjudgments often occur. Methods to improve the selection of sensor specificity to target gases and to reduce the cross response of interfering gases have been the difficulty and focus in the gas sensing area.

**Fig. 1:**
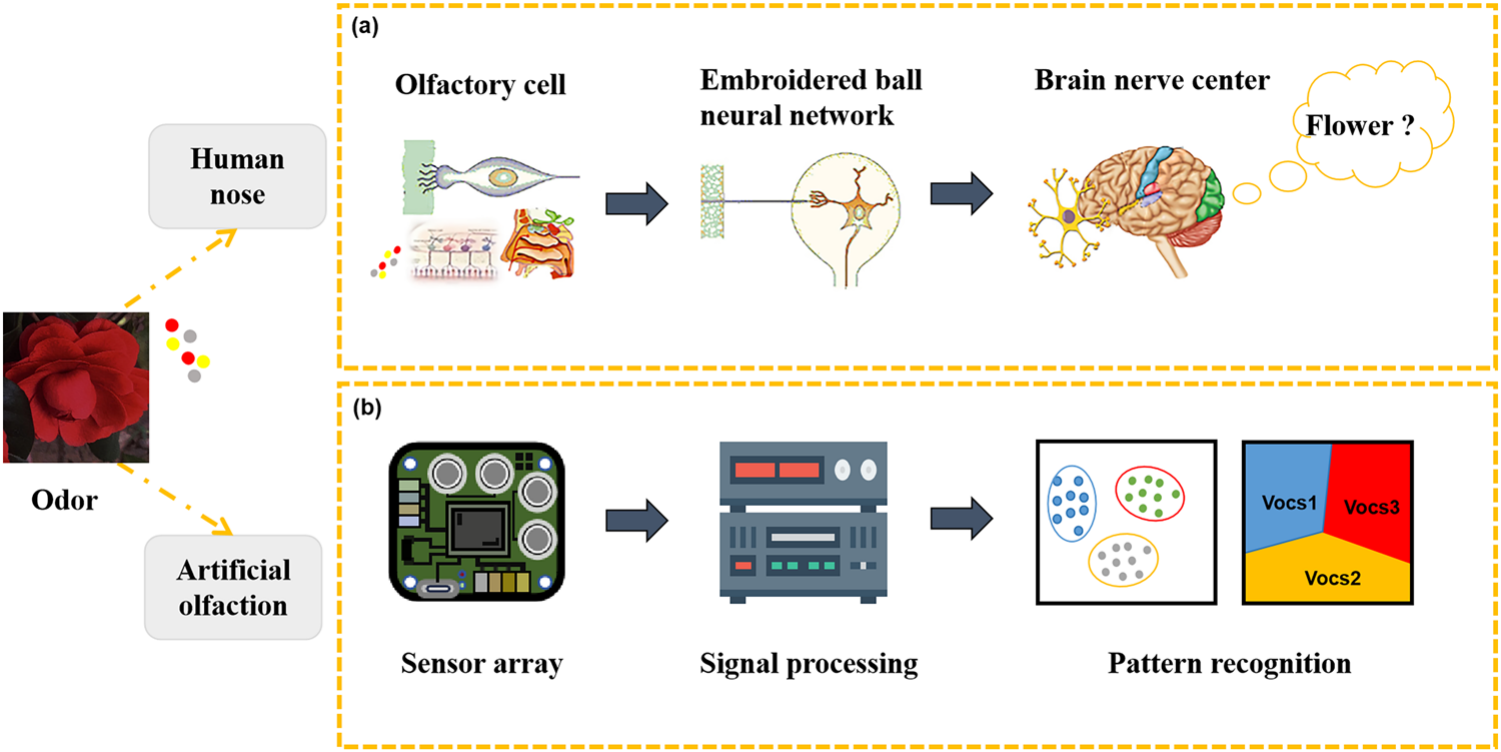
Process diagram of olfactory recognition. **a** Human olfactory mechanism. Reproduced with the permission from Ref. [260], Copyright Wiley 2019. **b** Artificial olfactory mechanism

An artificial olfactory system based on multi-sensor fusion technology which mimics the human olfactory system can overcome this problem. Figure 1a illustrates the intricate mechanism of the human olfactory system. A multitude of olfactory neurons within this system are specifically drawn to odor molecules, initiating the process of olfaction. When activated in combination with odors, complex odor fingerprints form and are sent to the cerebral nervous center for analysis. Figure 1b shows the schematic diagram of the artificial olfactory mechanism, which mainly consists of sensor array, data processing, and pattern recognition. Compared to the structure of the human olfactory system, a sensor array consisting of different gas sensors with cross-sensitive properties acts like olfactory neurons, which are sensitive to multiple odor molecules. Both of them convert chemical information about odors into electrical signals. The processing of response data bears a functional resemblance to the integration and amplification of electrical signals within the olfactory bulb. Meanwhile, the training and verification process of pattern recognition methods simulates the processing, learning, memory, and recognition of odor information in the human cerebral nerve center.

The multi-sensor array combined with pattern recognition methods has the potential to solve the problem of cross-sensitivity in gas sensors, realize the intelligent analysis of target gas, and further develop into a portable and real-time analysis system. An electronic nose (E-nose) is an intelligent device to realize gas recognition in artificial olfaction. Gas identification covers the detection, identification, and quantitative analysis of gas components through various sensors or instruments, and the use of pattern recognition algorithms for data analysis. The chemical memristor-based gas sensor designed by Chun et al. [19] realizes fast response, short recovery time and lagging gas response at room temperature without any additional equipment and circuits, which promotes the bionic technology of creating artificial intelligence systems. Cho et al. [20] designed a gas sensor based on a single micro-LED embedded light-activated, which uses time-varying lighting to achieve quantitative and qualitative analysis of complex gases. This method can effectively reduce the cost, space and power consumption of manufacturing electronic noses. Wang et al. [21] made a greater contribution, they integrated gas sensors on nanotubes, each chip can address 10,000 sensors separately, and can effectively distinguish 24 kinds of mixed gases at the same time, which greatly promoted the development of bionic olfactory technology.

**Fig. 2:**
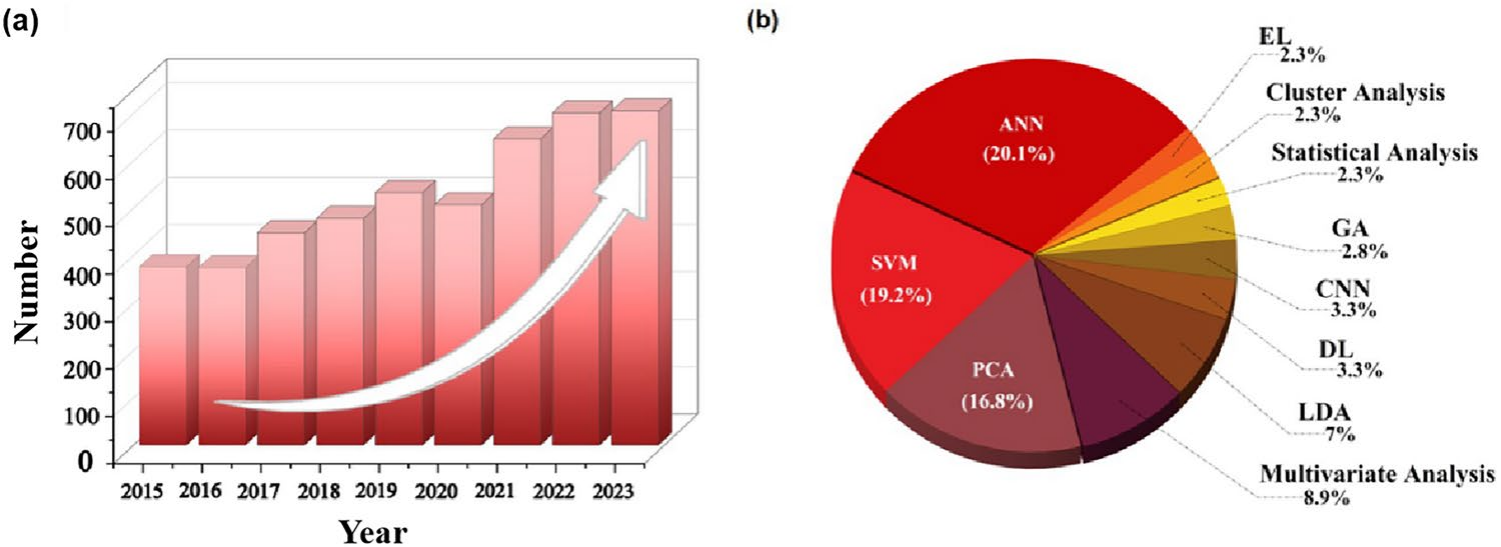
Research investigation based on gas pattern recognition. **a** The number of research papers on gas identification over the last decade. **b** The usage frequency of diverse pattern recognition methods (Topic = (electronic nose) and Publication Years = (2018‒2023) and Document Types = (Article) and Languages = (English). The initial 1000 papers are generated based on correlation, with titles and abstracts extracted using pattern recognition methods that filter out keywords with less than 15 occurrences)

In order to better understand the current research content and research status of gas identification, surveys were conducted. As depicted in Fig. 2a, the number of research articles on gas identification has been increasing annually over the past 10 years. A thorough search was conducted in Web of Science database, focusing on gas identification research using various pattern recognition methods between 2018 and 2023. The top 1000 research papers were selected based on their correlation. Among them, gas recognition research based on artificial neural networks (ANN) accounted for the highest proportion at 20.3%, followed by support vector machine (SVM) and principal component analysis (PCA) algorithms at 19.2% and 16.8%, respectively. In addition, other common methods are also included, such as linear discriminant analysis (LDA), convolutional neural network (CNN), deep learning, genetic algorithm (GA), and ensemble learning that integrates the results of multiple learners and some other analysis methods. The distribution of specific pattern recognition algorithms is depicted in Fig. 2b. Therefore, exploring more accurate and efficient pattern recognition methods is the principal research direction for solving gas cross-sensitivity.

It is important to note that different pattern recognition algorithms have different applicability. Pattern recognition algorithms applied in gas recognition are typically categorized into supervised and unsupervised learning. The supervised learning involves constructing a model based on known sample features or attributes to classify or predict unknown samples, including LDA, partial least squares regression (PLSR), SVM, random forest (RF), and K-nearest neighbor method (K-NN), while unsupervised learning can be further divided into three categories: PCA, cluster analysis, and neural network-based algorithms.

In specific studies, an appropriate pattern recognition algorithm selection is crucial to improve the gas recognition performance of the sensor array, as it can lead to better results and simplify the analysis process. For instance, PCA is a commonly used data dimension reduction method, which enables faster processing of unknown data in classification and regression tasks, thereby reducing the detection time. Saidi et al. [22] employed PCA to classify the output signal of the gas sensor into four health states: chronic kidney disease, diabetes mellitus, healthy subjects with high creatinine, and healthy subjects with low creatinine. Tohidi et al. [23] conducted a study comparing the recognition accuracy of milk samples using LDA and PCA load analysis. They found that PCA load analysis had higher accuracy in detecting three types of adulterations (formalin, hydrogen peroxide, and sodium hypochlorite) in milk, even at concentrations as low as 0.01%. However, it should be noted that when analyzing a large number of volatile organic compounds (VOCs), feature interpolation may occur in the PCA results, potentially affecting sensor performance. In such cases, nonlinear techniques like ANN algorithms are more suitable. Zarezadeh et al. [24] employed 7 pattern recognition algorithms to detect adulteration in olive oil samples, and the accuracy of ANN was the highest, reaching 95.51%. It is worth mentioning that the selection of model parameters based on the neural network also plays a crucial role in sensor performance, making it a challenging task to construct an appropriate neural network model. Of course, in addition to the classical neural network algorithm, many new methods have been proposed in recent years, and their fields of concern are more extensive. Sung et al. [25] proposed a data-centric method based on feature maps to implement a standardized artificial olfactory system. Experiments on complex mixed gases and automobile exhaust show that the method is effective and applicable for gas classification in various deep learning architectures.

Current reviews in the field of gas sensing primarily focus on sensor hardware design (e.g., reviews [26–28]), applications of gas sensing (e.g., review [29]), or provide introductory overviews of machine learning algorithms without detailed theoretical explanations (e.g., review [30]). However, there is a lack of systematic summarization regarding the challenges faced in gas sensing pattern recognition revealed through specific applications, and the principles, difficulties, and future directions of machine learning methods in addressing sensor cross-sensitivity. This review provides a comprehensive introduction to the pattern recognition algorithms based on chemiresistive gas sensor arrays, including the classification of algorithms, the working principle, characteristics, and the applicable gas detection range of different algorithms. The review also highlights the latest advances in the application of these algorithms in gas detection and demonstrates their application scenarios in three important domains including food safety, environmental protection, and medical diagnostics. Finally, the challenges and prospects of pattern recognition methods are discussed.

## Origins of Cross-Sensitivity

**Fig. 3:**
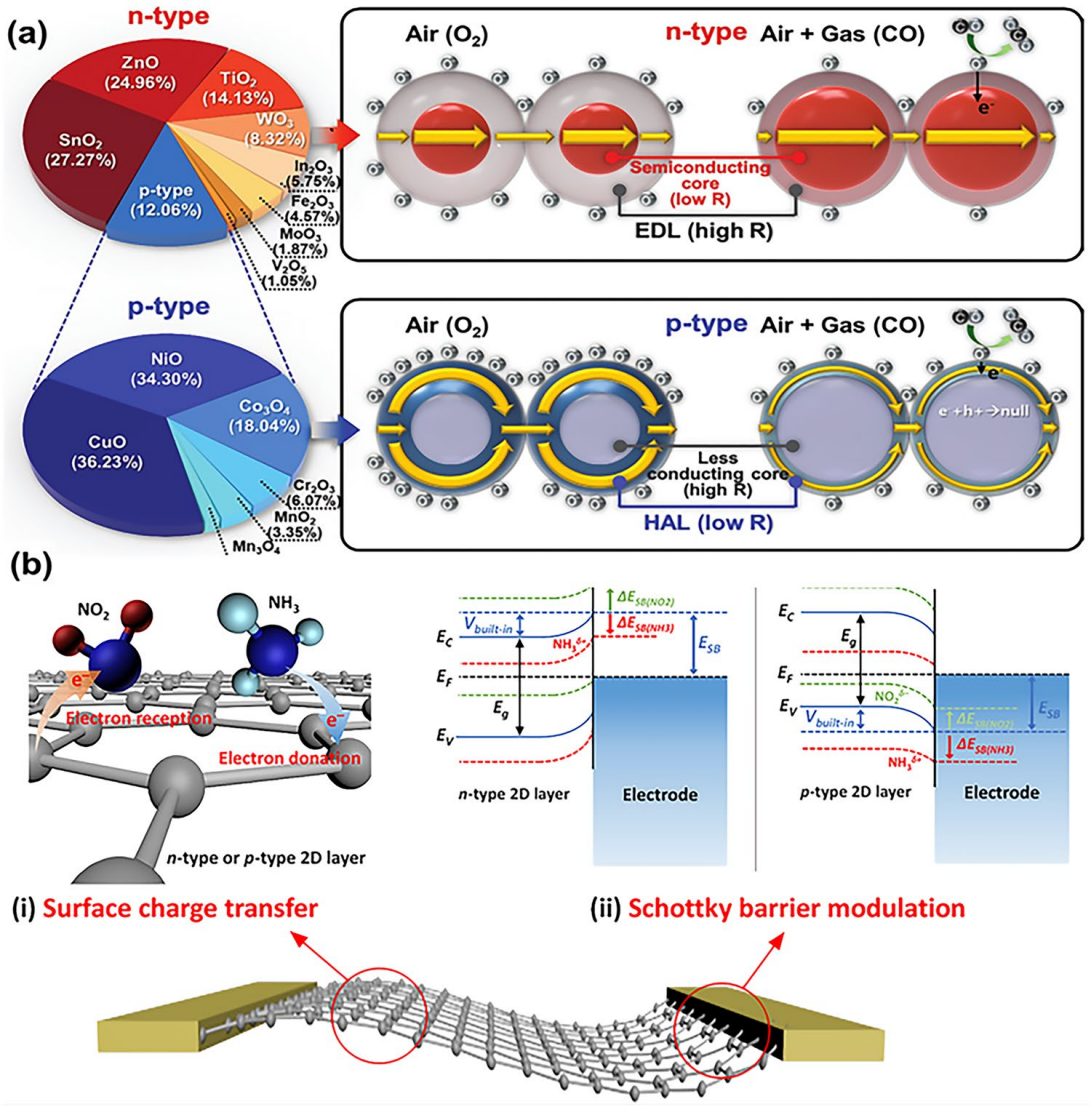
Schematic illustration of gas sensing mechanisms of different materials. **a** MOSs material. Reproduced with the permission from Ref. [261], Copyright Wiley, 2020. **b** 2D layered nanomaterials. Reproduced with the permission from Ref. [262], Copyright Springer, 2018

The performance of chemiresistive sensors is determined by their sensing mechanism, which is also responsible for their cross-sensitivity. For resistive-type metal oxide semiconductor (MOS) based sensors, one of the widely recognized sensing mechanisms is the oxygen adsorption model, which has been extensively studied. According to this model, when the MOS gas sensor is exposed to air, oxygen is adsorbed onto the metal oxide surface as stable free adsorbed oxygen ions (O_2_^−^, O^−^, O^2−^) [31] due to the high electron affinity of oxygen. The process of oxygen adsorption in oxide semiconductors leads to the formation of different electronic core–shell structures in n-type and p-type materials [32]. In n-type semiconductors, an electron depletion layer (EDL) is formed, while in p-type oxide semiconductors, a hole accumulation layer (HAL) is formed (Fig. 3a). When the target gas reacts with the adsorbed oxygen on the surface of sensitive materials, a redox reaction occurs, causing a change in the thickness of the depletion or accumulation layer. This change is reflected in the sensor resistance at a macroscopic level. However, any active gas can undergo an oxidation or reduction reaction with the sensor-sensitive layer, leading to a change in the resistance or conductivity of the gas-sensitive layer [33]. This phenomenon is the main reason for the cross-sensitivity of most MOS sensors to various gases and VOCs, which significantly limits their applications as gas-sensing materials.

However, for other materials, such as carbon-based materials, MXenes, and other typical one-dimensional or two-dimensional materials, the oxygen adsorption model is not applicable. Their sensitive properties primarily depend on the charge transfer mechanism, that is, the physical adsorption of gas molecules by the material through the Van-der-walls or the donor–acceptor interaction. Having more adsorption sites on the material or higher binding energy between the sensing material and the target gas is conducive to the detection of gas molecules. When gas molecules are exposed to the surface of nanomaterials, oxidizing gas molecules (e.g., NO_2_) tend to extract electrons from the nanomaterials. While reducing gas molecules (e.g., NH_3_) provide electrons to the nanomaterials. As shown in Fig. 3b, surface charge transfer can generally occur at the surface through direct carrier exchange between the adsorbed gas and the nanomaterial. For n-type nanomaterials, a decrease in conductivity is typically observed when the sensing layer is exposed to oxidizing gas molecules. Unlike oxidizing gases, reducing gases increase the conductivity of the n-type sensing layer. For p-type nanomaterials, opposite conductivity changes occur for oxidizing and reducing gases. In fact, most of the research is done on very complex materials. For these composites, different heterojunctions are formed between different materials (p–n, n–n, or p–p junctions). For p–n heterojunctions, depletion regions will occur at the interface due to the transfer of electrons from a high-energy conduction band state (for n-type semiconductors) to a low-energy valence band state (for p-type semiconductors). For n–n or p–p heterojunctions, carriers will flow from the high Fermi level material to the low Fermi level material until the Fermi level reaches equilibrium. This will lead to the formation of depletion and accumulation layers on the surface of the high energy state and low energy state, respectively, which has a great influence on the gas-sensitive performance. The construction of the heterojunction can significantly affect the base resistance of the sensor. The formation of EDL significantly increases the base resistance, while HAL leads to a decrease in the base resistance. In the reactive gases environment, when gas molecules interact with sensitive material, capturing electrons from gas molecules or supplying electrons to gas molecules results in a change in the EDL or HAL width. The transfer of a small number of electrons in the heterojunction leads to a dramatic change in the conductivity of the material compared to a single material. This change is macroscopically reflected in the enhanced response of the sensor to the gas molecules. In addition, heterojunctions have stronger dangling bond oxygen vacancies, more active sites, and stronger catalytic activity. Multi-nanocrystalline junctions have more conducting electron transfer channels compared to single nano-junctions. Therefore, fast electron transfer in multi-nano-junctions leads to fast detection times. Whether based on charge transfer mechanism or electron movement in heterojunctions, sensitive materials can trap ambient gas molecules, resulting in cross-sensitivity.

Extensive research has been conducted to improve the selectivity of gas sensors [34–36]. The response of the sensor to certain gases can be enhanced through modifications such as stoichiometric ratio regulation, metal cation doping, and noble metal doping. However, it remains a challenge to meet the requirements for gas composition sensing in complex environments [37]. The combination of sensor array pattern recognition methods and artificial intelligence technology has gained significant attention in gas sensing analysis.

## Pattern Recognition Methods

The artificial olfactory system comprises a gas sensor array and artificial intelligence to achieve qualitative and quantitative analysis of gases. Pattern recognition, as a crucial component of artificial intelligence, analyzes and processes the signal data output by multiple sensors to recognize and classify different gases, thereby enhancing the accuracy of gas recognition. Various pattern recognition methods have been integrated into engineering olfactory sensor technology to mimic natural olfactory detection [29, 38, 39]. In recent years, deep learning methods based on neural networks have emerged, offering more possibilities for model construction and optimization. For example, Lee et al. [40] developed a principal odor map (POM) using graphical neural networks. This POM can link physical characteristics with perceptual characteristics. The POM successfully encodes a generalized map of the relationship between structure and odor on several odor prediction tasks. The emergence of this method provides a meaningful idea for odor digitization. This part primarily focuses on introducing classical algorithms and neural network algorithms to gain a better identification of chemiresistive sensor responses.

### Classical Algorithm

Many classical algorithms have been applied in chemiresistive sensor arrays to process gas response signals, including LDA, PLSR, SVM, PCA, K-NN, RF, hierarchical cluster analysis (HCA), and so on. Here, the mechanisms and properties of these algorithms are discussed. Table 1 lists the advantages and disadvantages of classical pattern recognition algorithms and their applicable scope.

**Table 1:** Comparison of classical pattern recognition algorithms

Algorithm	Description	Advantages	Disadvantages	References
LDA	Reduce the data dimension while maximizing the separability between different categories	Simple and fast Both classification technology and dimensionality reduction technology	Sensitive to outliers Not suitable for non-Gaussian distribution samples A linear relationship is needed	[263]
PLSR	Relationships between input variables and responses by extracting latent variables that have the best predictive power	Suitable for multivariate multiple linear regression analysis Useful in the case of large differences in data Feature selection and classification can be combined simultaneously	Limited to linear regression analysis The local structure of the data is not retained	[186]
SVM	Maximizes the margin between different classes of data points	High stability for high-dimensional space No need for a large number of samples No use of covariance information	Sensitive to outliers	[55]
PCA	Capture the main variation information in the data	Effective dimension reduction Remove redundant information Convenient visualization	Limited to a linear relationship Sensitive to data distribution	[264]
K-NN	Classifies new cases based on a majority vote of its K nearest neighbors in the feature space	Easy to implement Strong adaptability Fewer hyperparameters Does not occupy the training period	Require for large data size Perform poorly in high-dimensional data Easy to appear over-fitting Sensitive to outliers	[265]
RF	Construct multiple decision trees and synthesize their prediction results	Effectively deal with outliers Not easy to over-fitting Strong anti-noise ability Wide range of data types Few hyperparameters	Speed is affected by the decision tree High computational cost	[266]
DT	The data is segmented by recursively selecting the optimal feature	Nonparametric method Processing a wide range of data types Not require feature scaling Easy visualization for tree diagrams	Replication problem Shortsightedness instability Fragment problem Dimension limitation Not suitable for large data sets	[69]
HCA	The data set is divided into a hierarchical structure gradually aggregated by similarity	No need to pre-customize the number of clusters Fewer restrictions Hierarchical relationship of classes	Great demand for time and space Vulnerable to the impact of merging or splitting points	[267]

#### Principal Component Analysis

**Fig. 4:**
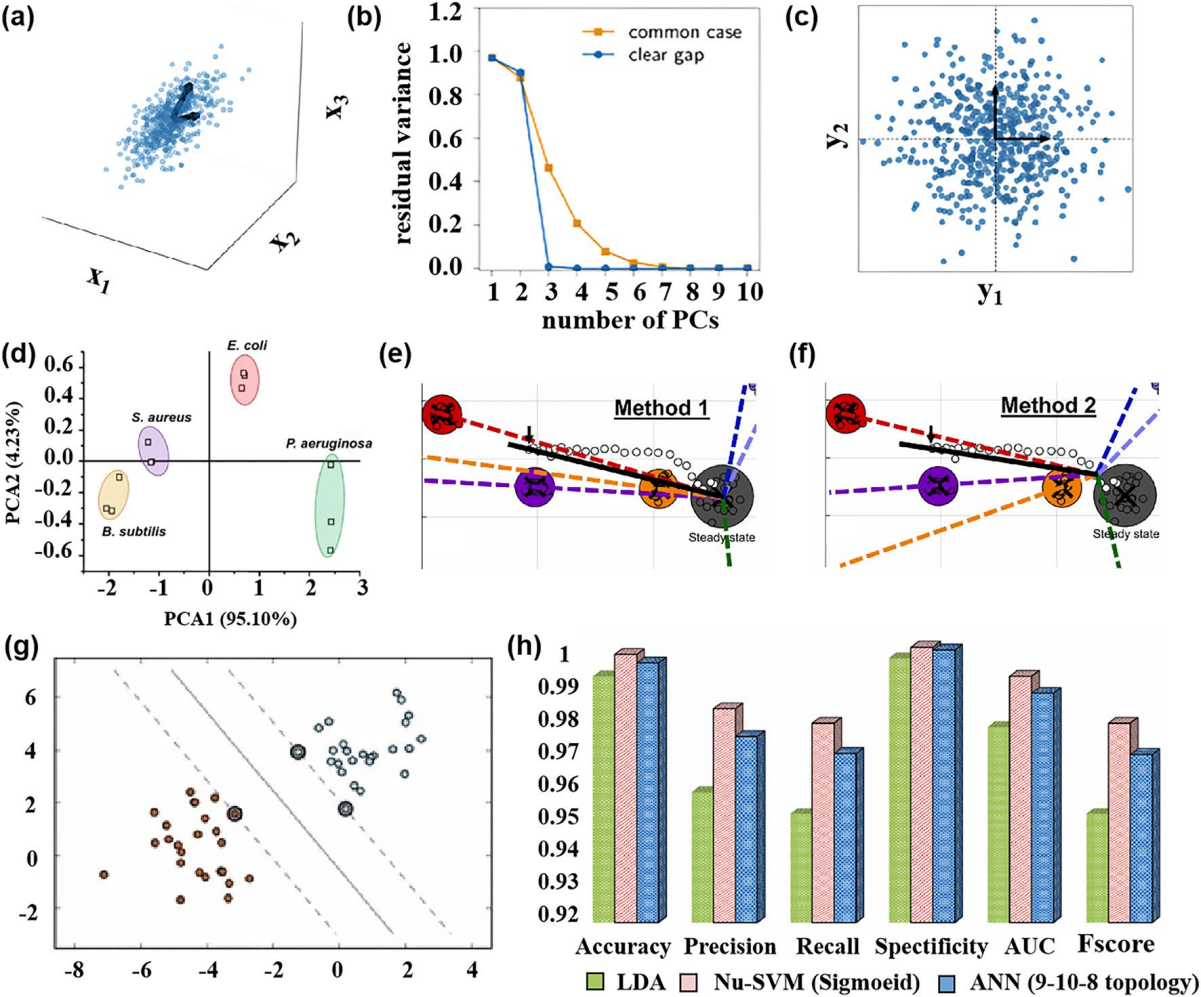
**a** Two-dimensional linear manifold representation of high-dimensional space. **b** Features of a covariance matrix of the data, the blue line represents a clear gap and the orange curve represents the common case. **c** Two-dimensional representation after PCA dimensionality reduction. Reproduced with the permission from Ref. [43], Copyright American Chemical Society 2021. **d** PCA results to distinguish different species of bacteria. Reproduced with the permission from Ref. [46], Copyright Springer 2020. **e** Steady-state gravity in the training data is used as the discrimination model of the target gas in the direction of the LD fraction of the reference point to the test data. **f** LD fraction that first leaves the steady-state region is used as the discrimination model of the target gas in the LD fraction direction from the reference point to the test data. Reproduced with the permission from Ref. [53], Copyright Elsevier 2023. **g** SVM of the kernel function is used to find the largest hyperplane between different classes. Reproduced with the permission from Ref. [55], Copyright Springer 2019. **h** Three methods were used to classify the average performance parameters of essential oils under 8 drying methods. Reproduced with the permission from Ref. [58], Copyright Elsevier 2023

For dimensionality reduction in complex models with multiple variables [41, 42], the mainstream method used is PCA, which is an unsupervised method for feature extraction. The PCA creates a new dimension set by associating the initial dimension and the compressed variance (Fig. 4a–c). It projects the two-dimensional data set into one dimension based on the features of a covariance matrix [43]. The new data set after PCA dimensionality reduction retains 60%–99% of the initial data, making it suitable for sensor signal extraction and gas monitoring applications [44]. However, the limitation of the PCA algorithm is that it may not effectively separate subsets if the input data set is small. The selection of feature vectors, which are used to generate new feature spaces, is the key step in PCA. These feature vectors correspond to the array’s response to a given analyte, allowing the user to determine the array’s sensitivity to specific gases in its external environment.

Furthermore, PCA is commonly used for data classification. For example, Tang et al. [45] applied PCA to extract principal components from an odor fingerprint information database of pesticide residues in apples, detected by MOS sensors, resulting in the successful detection of all apple samples. Shauloff et al. [46] developed a new artificial nose based on electrode-deposited carbon dots combined with machine learning methods to predict individual and mixed gases. Figure 4d shows the classification of bacterial species based on the first two principal components, we can observe that there is no overlap between different species.

In gas recognition, PCA has advanced through integrating multimodal feature extraction and sensor data fusion. Recent research has enhanced gas recognition systems with refined PCA variants and synergistic approaches involving deep learning and reinforcement learning [47, 48]. As sensor technology evolves, PCA is poised to impact large-scale data processing for applications like real-time monitoring and intelligent control systems. However, it faces challenges such as noise, sensor drift, nonlinear relationships, heterogeneous data integration, high-dimensional data with limited samples, interpretability, and non-steady-state conditions.

#### Linear Discriminant Analysis

LDA is a supervised feature extraction method that has been extensively studied in the fields of statistics and pattern recognition. It aims to find the optimal transformation of features by training samples and their corresponding class labels. This allows for the extraction of discriminant features that can represent multiple types of objects [49]. The main idea of LDA is to maximize the distance between data belonging to different classes and minimize the distance within groups after projection. LDA is closely related to regression analysis, such as linear regression [50] and logistic regression, making it suitable for solving regression and classification problems. For gas response signals from multi-sensor arrays, LDA can be used not only for data preprocessing but also for the classification of different gases.

Yin et al. [51] found that after applying LDA to the preprocessing of a high-dimensional (greater than 70) gas feature dataset, only five-dimensional features are needed for gas mixture classification. This reduction in the complexity of the model not only shortens the calculation time but also maintains classification accuracy. This indicates that LDA has achieved remarkable success in explaining data variance and sample classification. Aghdamifar et al. [52] used a variety of methods to classify coffee beans. The R^2^ of the LDA model was as high as 0.9714. Itoh et al. [53] improved the LDA method to analyze multiple semiconductor sensors to distinguish target gases in complex environments. Figure 4e, f show the improved LDA algorithm. Method 1 is based on the steady-state gravity of the training data set as the direction division. Method 2 is based on the linear discriminant score of the data set that leaves the steady-state region first.

LDA creates a classifier by training samples, but its main disadvantage is that it requires a large sample size. This becomes problematic for high-dimensional sensors, where the number of collected samples is lower than the sensor dimension, resulting in an unstable prediction matrix. To address this issue, a method called “merging covariance” has been proposed to calculate the prediction matrix. However, this method can still cause sharp fluctuations in the LDA results, especially when the sample group is small, which is known as overfitting.

In gas recognition, LDA is used for dimensionality reduction and classification, excelling in extracting key features from sensor response data and distinguishing between different gas types. Nonetheless, LDA faces challenges such as handling class imbalance, managing within-class variance, the limitations of the linear separability assumption, and the issue of high-dimensional data with limited samples.

#### Support Vector Machine

SVM is a linear classifier designed to find a separating hyperplane in feature space, optimizing through iterations to maximize classification performance. It aims to partition the training data set while maximizing the geometric margin. It uses a suitable kernel function to effectively handle the inner product operations in high-dimensional space. This approach enables the handling of nonlinear classification problems [54]. Figure 4g shows the geometric view of the kernel. In this space, SVM applies the kernel function to find the hyperplane that supports the largest gap between nonlinear separable data [55]. The choice of the kernel function is crucial in constructing a high-performing SVM. This involves selecting the kernel function type and determining the relevant parameters. Commonly used kernel functions include linear, polynomial, Gaussian, and Sigmoid. Nonlinear SVM is based on the use of linear SVM in various applications. For instance, it can be applied to classify cerebral imaging based on morphological features or identify possible fractures in bones using anatomical structure images (such as X-rays) [56]. SVM is considered a promising classical learning method for solving both classification and regression problems [57].

For the output signal of the sensor array, SVM is often used as an optimizer because it can classify different categories through multiple iterations. Compared with PCA and LDA, SVM has the advantage of not requiring large data sets and covariance information. Its predictive ability can be improved through multiple iterations. SVM was used to train the radial kernel C-classification SVM and successfully classify all 8 aromas by inputting 14 data sets. This demonstrates that SVM can effectively solve the problem of cross-interference and improve the resolution of E-nose. Rasekh et al. [58] used LDA, SVM and ANN algorithms to classify the essential oil of spearmint under eight drying methods. As shown in Fig. 4h, the accuracy and specificity of all models were greater than 0.99, while the classification performance of the Nu-SVM method using the Sigmoid kernel function was the best.

Researchers have significantly enhanced the performance of SVM in gas recognition by optimizing kernel selection, parameter tuning, and feature engineering methods, effectively addressing complex gas data patterns and classification tasks [59]. Furthermore, the integration of SVM with deep learning techniques such as CNN has emerged as a critical strategy for improving classification accuracy and robustness. Looking forward, SVM is poised to continue playing a pivotal role in gas recognition, particularly in tackling challenges posed by nonlinear and high-dimensional data, as datasets grow larger and algorithmic optimizations deepen. In gas recognition, SVM exhibits versatility and efficiency in data separation, yet its computational complexity may pose challenges when dealing with large-scale datasets.

#### K-Means and Hierarchical Cluster Analysis

Clustering analysis is a prevalent data analysis technique employed to group samples based on the inherent relationships within the data [60]. It can be considered as a form of dimensionality reduction. k-means clustering [61] and HCA are common algorithms for clustering.

**Fig. 5:**
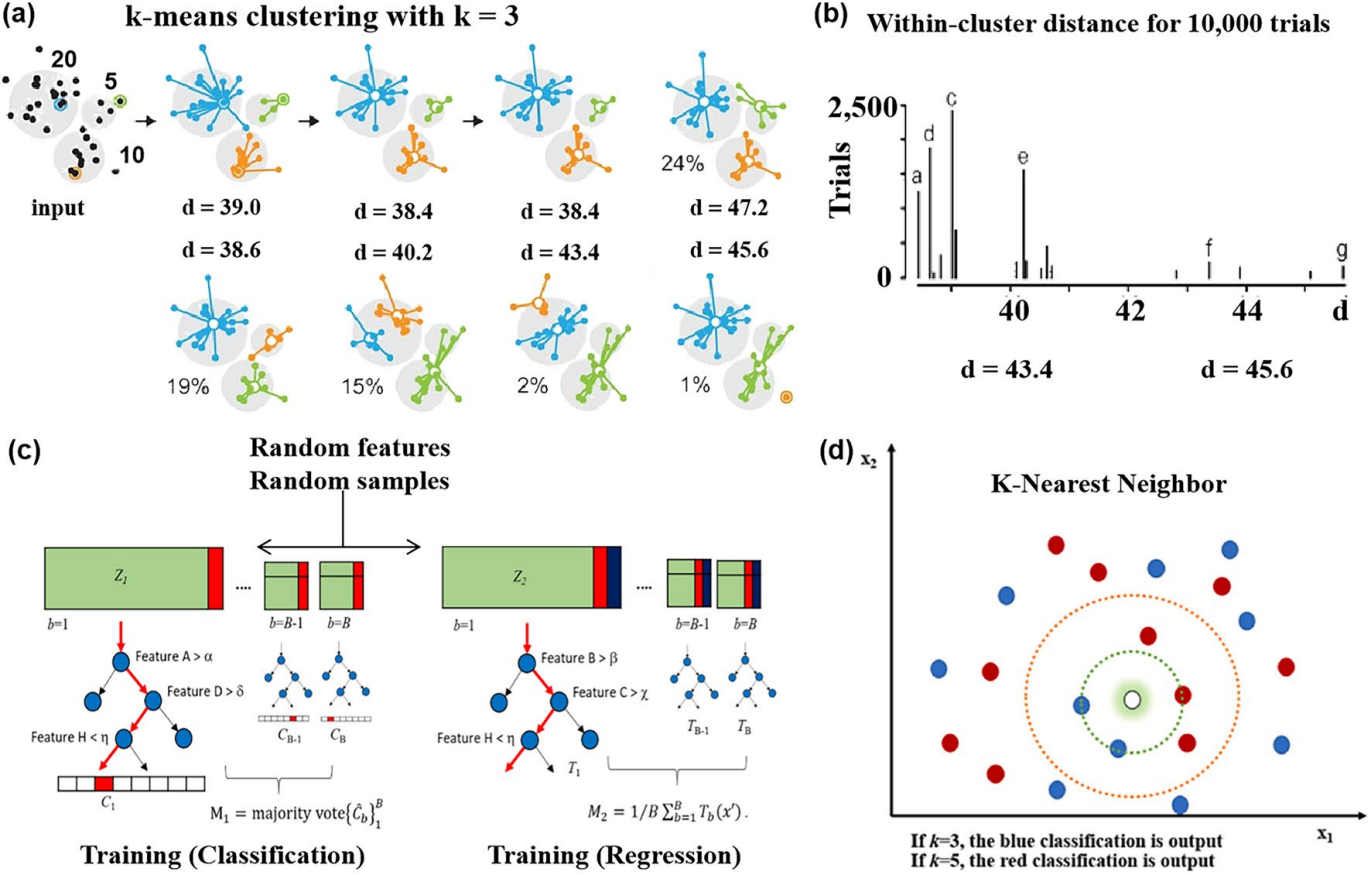
**a** k-means clustering experiment, k = 3, the number of data points are 20,10, and 5, respectively, and d is the radius of the circle. **b** Represents the intra-cluster distance histogram of 1000 experiments. Reproduced with the permission from Ref. [63], Copyright Nature 2017. **c** Using HCA to distinguish three asthma VOCs. Reproduced with the permission from Ref. [74], Copyright Elsevier 2020. **d** K-NN model diagram. Reproduced with the permission from Ref. [77], Copyright Elsevier 2023

K-means clustering is a commonly used method in clustering analysis. The main objective of this algorithm is to minimize the loss function by iteratively finding a partition scheme of k clusters [62]. Unlike hierarchical clustering, the k-means algorithm requires the prior determination of the number of clusters [63], as depicted in Fig. 5a, b. The k-means algorithm separates a two-dimensional dataset into three clusters. Initially, three data points are randomly chosen as the initial centroids in three adjacent regions. The algorithm then calculates the distance between each data point and the centroid, assigning each point to the nearest centroid. Next, the centroids are recalculated within each group, and the process is repeated until the centroids and clusters remain unchanged. Figure 5b illustrates the histogram of the total distance in the cluster after 10,000 experiments. The k-means clustering method has been extensively studied in partitioning techniques and applied in various areas [64–66]. Licen et al. [67] utilized a multi-sensor array combined with the k-means clustering algorithm to generate different datasets for monitoring ambient air in an industrial area in southern Italy.

HCA is an unsupervised algorithm used for clustering analysis as well as data classification. In the process of implementing the algorithm, each datum plane is identified as a separate cluster, and then the Euclidean Distance from each cluster to the datum plane is calculated, then the most similar clusters are combined in order. This process is repeated until the best cluster is formed.

Hidayat et al. [68] combined HCA with the permutation feature importance method to identify highly correlated features. This approach improved the analysis of multi-sensor array data for coronavirus disease 2019 (COVID-19) detection, while also reducing the number of sensors required.

HCA and K-means are widely used in gas recognition for their simplicity and effectiveness in clustering similar sensor response patterns. However, HCA’s sensitivity to noise and inability to dynamically adjust its hierarchical structure limit its practicality for modern gas recognition systems requiring continuous adaptation to new data. In contrast, advanced machine learning algorithms like SVM, RF, and CNN offer superior performance and flexibility in handling complex gas data, gradually replacing traditional clustering methods in this domain. However, they face unique challenges specific to gas data analysis. K-means is sensitive to initial cluster centroids and requires a predefined number of clusters, which can be difficult to determine accurately in complex gas datasets with varying response patterns. On the other hand, HCA's performance can be impacted by the choice of linkage criteria and the challenge of interpreting dendrogram structures in the context of gas sensor data.

#### Decision Tree and Random Forest

Decision Tree (DT) is a tree-based method commonly used for classification and regression tasks. It divides the training set into subsets based on hierarchical decisions about features. DT consists of nodes and leaves, where nodes are used to split the data into smaller partitions until no further partition is needed, and leaves represent the final partition [69]. DT offers several advantages, such as being a non-parametric method capable of processing various types of input data and achieving high prediction performance even with original errors or missing values. However, one disadvantage is that DT is oversensitive to training sets, irrelevant attributes, and noise [70], which can result in overfitting when the tree is deep.

Herrmann et al. [71] utilized a decision tree classifier to evaluate a sensor database, successfully observing dynamic changes in plant respiration patterns. They accurately identified water deficiency in soybean plants with a precision rate of 94.4%. Future investigations should be conducted under controlled conditions to enable early detection and monitoring of stress levels.

DT excels in handling large-scale data due to their efficient tree structure, which ensures fast data processing and low memory consumption, making them highly suitable for high-dimensional and big data analysis tasks in gas recognition. Furthermore, when combined with other ensemble learning algorithms such as RF and Gradient Boosting Trees, DT can effectively reduce overfitting and significantly enhance classification accuracy and stability. In the future, as Internet of Things (IoT) devices continue to collect vast amounts of environmental data, the rapid classification capabilities of DT will make them an ideal choice in this context.

RF is a nonlinear statistical ensemble method that utilizes multiple decision trees to solve the problem [72]. It acts as a supervised learning classifier, aggregating the results of these decision trees to produce a single result. This approach helps prevent overfitting and reduces errors. Randomness plays a significant role in RF. Firstly, each decision tree randomly selects and retrieves N training samples from the training set as its training set. Secondly, at each node, a random subset of feature dimensions (m < M) is chosen from the total number of features (M) in each sample, and the best split is determined from these variables [73]. As shown in Fig. 5c, the random forest model constructed by Kim et al. [74] to effectively capture the response pattern of the sensor array is used to classify the test samples.

The classification capabilities of RF rely on two sources of randomness, which help prevent overfitting and enhance noise resistance. RF offers several advantages over other machine learning methods, including low complexity, fast computation, and a low overfitting rate. In various applications, such as food quality control and disease diagnosis, RF has proven to be effective. Du et al. [75] successfully predicted the overall maturity, soluble solids content, and firmness of kiwifruit using MOS E-nose combined with RF.

DT is favored for its simplicity and interpretability, capable of handling nonlinear relationships in gas sensor response patterns. On the other hand, RF excels in managing high-dimensional data and noise, leveraging ensemble learning for improved accuracy. However, RF may encounter challenges when dealing with imbalanced datasets, requiring careful handling of class distributions to maintain robust performance in gas recognition applications.

#### K-Nearest Neighbor

K-NN is a supervised learning method commonly used in classification and regression. The working principle is to divide the feature vector space using the training data and use the division results as the final algorithm model [76]. Figure 5d shows that K-NN uses different distances to retrieve valid data sets to achieve classification [77]. This allows data to be classified based on labels. If the input data is unlabeled, each feature of the unlabeled data is compared to the corresponding feature of the data in the sample set. The classification label of the most similar data (nearest neighbor) in the sample is then extracted.

The K-NN classification prediction process involves finding a set of k vectors closest to the input vector x in the training data set. The category of x is then predicted based on the category with the highest frequency among the k samples [78]. The value of k in the K-NN algorithm determines the number of nearest neighbors to consider. Various distance metrics, such as Minkowski Distance, Euclidean Distance [79], Manhattan Distance, Chebyshev Distance, and Cosine Distance, are commonly used to calculate the distance between predicted targets. In the study by Sironi et al. [80], two E-noses monitored the ambient air near a factory for four consecutive days. K-NN algorithm was employed to classify the odors and the test results were compared with the residents’ perceptions. It was found that with appropriate training, the E-nose achieved 78% accuracy in both qualitative and quantitative recognition of odors.

Although the K-NN classifier is simple and practical, finding the best K value is challenging. To address this issue, several scholars have proposed alternative methods for dynamically selecting the optimal k value. Manocha and Girolami [81] propose a probabilistic nearest-neighbor method to infer the optimal k value by determining the number of neighbors.

The future development trends of K-NN in gas recognition will be centered around integrating ensemble learning methods, incorporating deep learning, optimizing computational efficiency, and implementing dynamic adaptation and adaptive learning strategies. K-NN is also widely employed in gas recognition tasks, valued for its simplicity and adaptability to diverse data distributions. Nevertheless, K-NN can be computationally intensive, especially with large datasets, and is sensitive to noise and irrelevant features present in gas sensor data.

#### Partial Least Squares Regression

PLSR is a multivariate linear regression method that combines PCA, canonical correlation, and multivariate linear regression [82]. In regression analysis, the presence of multicollinearity in the data can pose problems. Ordinary multiple linear regression is not effective in solving these problems, but PLSR can address them well. The working principle of PLSR can be understood as follows: First, the principal components U and V corresponding to multiple X and multiple Y are extracted using PCA. Then, the relationship between X and U along with the relationship between Y and V are analyzed using canonical correlation. Finally, by combining the principle of multiple linear regression, the relationship between X and V is obtained, subsequently, the relationship between X and Y is obtained. Overall, PLSR reduces the number of observable variables and extracts principal components for analysis to maximize the correlation between the observed and predicted variables. Therefore, the PLSR method is suitable for solving target prediction problems based on sensor array data.

Dong et al. [83] combined E-nose and electronic tongue (E-tongue) sensors with chemometric multivariate analysis to characterize and classify coffee varieties, in which, the PLSR model with a fusion data set was considered the best model for determining quality parameters. Khorramifar et al. [12] utilized PLSR to accurately simulate the relationship between odors of different types of potatoes, sugars, and carbohydrates.

PLSR is frequently employed in gas recognition for its ability to handle multicollinearity and extract relevant information from high-dimensional sensor data. However, it may suffer from overfitting when the number of predictors is larger than the number of samples.

### Neural Network Algorithm

Previously, classical algorithms such as SVM and LDA for gas pattern recognition were discussed. However, these analysis methods have limitations as they only consider the linear relationship of the sensor data in certain cases. For instance, PCA and LDA rely on linear correlation between dimensions, while LDA and SVM depend on linear separability [84]. Therefore, a neural network algorithm that mimics the processing mechanism of complex information in the human brain’s nervous system is considered a powerful analytical tool for handling nonlinear data.

Neural networks, a branch of machine learning, combine multiple linear regression models with input and activation functions to create a pattern recognizer. Hence, neural network-based algorithms are also seen as the most promising tools for addressing the issue of cross-sensitivity in sensor arrays [85, 86]. Furthermore, new neural network models have been proposed for processing sensor array response gas data. This section focuses on several neural network models, including parallel neural networks (PNN), feedforward neural networks (FNN), backpropagation neural networks (BPNN), extreme learning machines (ELM), and CNN etc. Table 2 lists the advantages and disadvantages of the neural network algorithm and the scope of its application.

**Table 2:** Comparison of different algorithms based on neural network

Algorithm	Description	Advantages	Disadvantages	References
PNN	Predict data based on Bayesian statistical approaches	Approach the Bayes optimal decision surface provided that the class probability density functions are smooth and continuous Fast training	Requires a good amount of data for training and test	[268]
FNN	The simplest type of ANN wherein connections between the units do not form a cycle	Easy to understand Easy to implement	Gradient disappearance or gradient explosion	[269]
ELM	Single-layer FNN using randomly assigned hidden nodes	Fast convergence speed Small training error Good generalization ability	Hidden layer node redundancy in model construction	[270]
CNN	Implement convolutional layers that automatically learn spatial hierarchies of features	Shared convolution layer high-dimensional data processing	Need to manually select features Need a large number of training samples	[98]
RNN	Connections between nodes form a directed graph along a temporal sequence	Processing sequence data Weight sharing Capture long-term dependence	High computational complexity Gradient disappearance or gradient explosion Hyperparameter sensitivity	[271]

#### Parallel Neural Networks

PNN is essentially a parallel algorithm that combines probability density and Bayesian minimum risk criteria. Under certain conditions, the decision surface generated by PNN closely approximates Bayesian optimal estimation. Structurally, PNN consists of four layers of neural networks: input layer, hidden layer, summation layer, and output layer. It can map any input pattern to any number of classifications. The application of the PNN analysis method to the study of gas response data based on sensor arrays has proved to be effective. Kalinichenko et al. [87] utilized PNN to classify the odor of sensor data and successfully achieved rapid analysis of sausage identification and adulteration. Researchers utilized PNN to analyze the air quality in different indoor and outdoor environments using four types of sensors: kitchen, living room, bar, and terrace [88]. They achieved classification accuracies of 98.22% and 99.47% when using two sensors and four sensors combined with PNN, respectively. This study provides valuable insights for the future development of low-cost devices to monitor air quality under various indoor and outdoor conditions, aiding in the early establishment of safety measures to prevent diseases.

PNN features a straightforward model structure that is easy to comprehend and implement. They handle data using a probabilistic framework, exhibiting robustness to noise and minor missing data. However, PNN may struggle with the curse of dimensionality when confronted with high-dimensional or feature-rich datasets, leading to significant computational overhead. Like many machine learning models, PNN typically requires substantial labeled data for training to ensure robust generalization and accuracy. Future advancements in PNN will focus on optimizing algorithms and structures, exploring synergies with other advanced technologies, crucial for enhancing performance in gas recognition and beyond. PNN is utilized in gas recognition for its capability to model complex nonlinear relationships and handle uncertainties in sensor data. Nevertheless, it may require substantial computational resources for training and inference.

#### Feedforward Neural Networks and Backpropagation Neural Networks

**Fig. 6:**
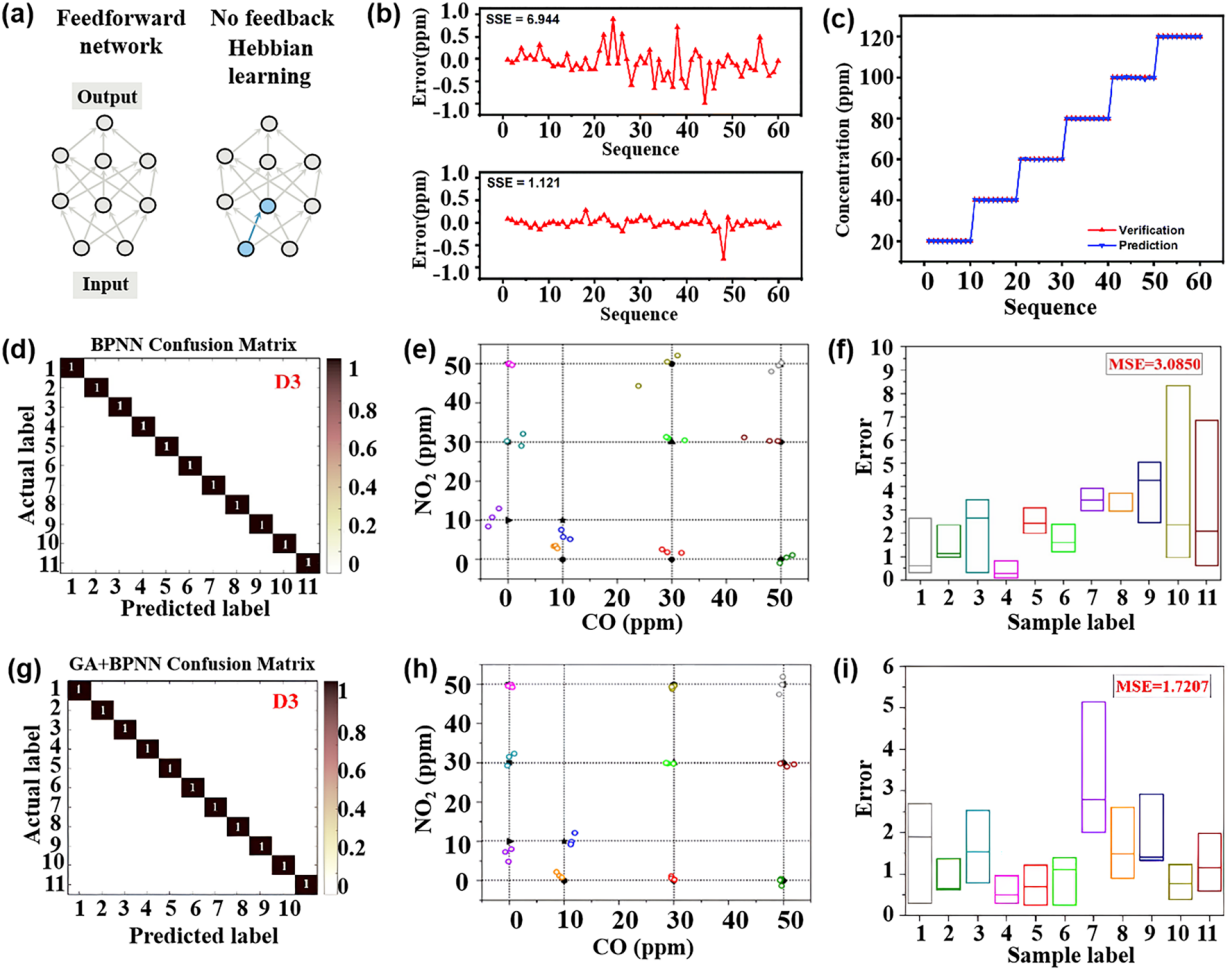
**a** Simple FNN. Reproduced with the permission from [84], Copyright Nature 2020. **b** The error between the validated concentration and the predicted concentration of the BP-based and ELM-based regressors. **c** Verified and predicted concentrations of the ELM-based model. Reproduced with the permission from Ref. [96], Copyright Royal Society of Chemistry 2021. **d**‒**f** Corresponds to the normalized confusion matrix, correlation performance, and predicted concentration box diagram of BPNN detection of CO and NO_2_. **g**‒**i** Corresponds to the normalized confusion matrix of GA plus BPNN for detecting CO and NO_2_, the correlation performance, and the predicted concentration box diagram. Reproduced with the permission from Ref. [107], Copyright Elsevier 2021

FNN is a commonly used algorithm in ANN. It consists of three layers: the input layer, the hidden layer, and the output layer. FNN is a one-way network where input information is transmitted from the input layer to the next layer of neurons until it reaches the output layer. The output results of each node in the hidden layer are determined using an activation function, such as the commonly used Sigmoid, hyperbolic tangent, or ReLU activation functions. Figure 6a illustrates a simple FNN. Depending on the number of hidden layers, the FNN can be classified as a single-layer network or a multi-layer network. The FNN is known for its simple structure, wide applicability, and strong nonlinear processing capability, making it suitable for solving classification and regression tasks.

BPNN is a multi-layer FNN that utilizes the back propagation learning algorithm [89]. The implementation process of BPNN involves activating the product of the data in the input layer and the initial weight using the activation function and then passing it to the hidden layer until the output layer is obtained. The activation function applied is Sigmoid, which enables the network to achieve any nonlinear mapping from input to output. The error between the output value and the expected value is then calculated using the loss function. The weights of each neuron in each layer are updated by a chain rule to minimize errors [30]. In back propagation, the vector error signal is transmitted backward along the original influence path of the neuron. However, in the brain, vector feedback can be transmitted in various ways, and vector transmission can be achieved through a separate network. As one of the most successful gradient descent methods for training neural networks [84], back propagation has found numerous applications in processing sensor signals. For instance, Jiang et al. [90] constructed a BPNN model to classify Jinhua dry-cured hams with an accuracy of 100%.

FNN and BPNN each have inherent strengths and weaknesses in the field of gas recognition. FNN processes data by sequentially passing signals through layers, offering a simple structure that is easy to implement and understand, but it may struggle with complex patterns and nonlinear relationships. BPNN, an extension of FNN, optimizes weights and biases using backpropagation, enabling it to handle complex nonlinear problems with strong learning capability and adaptability. However, BPNN training can encounter challenges such as gradient vanishing or exploding during training.

Looking ahead, the future development of these algorithms in gas recognition will focus on optimizing neural network architectures and algorithms to enhance their ability to recognize complex gas patterns. With advancements in deep learning technologies, FNN and BPNN will increasingly integrate with advanced models like CNN, aiming to improve the performance and robustness of gas recognition systems for real-time monitoring in diverse environmental conditions. FNN and BPNN both exhibit powerful pattern recognition capabilities in gas recognition, capable of handling complex nonlinear relationships. However, the training process may be slow and susceptible to local minima, necessitating careful parameter tuning for optimal performance.

#### Extreme Learning Machine

ELM is an algorithm used to solve single hidden layer neural networks. ELM is known for its high learning efficiency and accuracy [91]. During the ELM modeling process, the hidden layer randomly generates connection weight parameters and neuron thresholds using an infinitely differentiable function [92, 93]. ELM effectively solves the regularized least squares problem by combining the training error term, the regularization term of the output layer weight norm, and the Moore–Penrose generalized inverse matrix analysis method to determine the output weight between the hidden layer and the output layer. Overall, ELM offers advantages such as fewer training parameters, fast learning speed, and strong generalization ability [94]. In recent years, there have been advancements in the theory, application, and practical use of ELM. Zhu et al. [95] proposed an evolutionary ELM with a more compact network and faster training response speed. In an experiment by Wang et al. [96], backpropagation (BP) and ELM were used to establish prediction models for ethanol, respectively. As shown in Fig. 6b, the error oscillation amplitude based on the BP model and ELM model is in turn above and below. It can be seen that ELM has a more stable performance. Figure 6c is the comparison between the predicted concentration and the actual concentration of ethanol based on the ELM model, and there is no obvious deviation in different concentrations. ELM are characterized by their fast learning speed and ability to handle large-scale datasets efficiently. They operate by randomly initializing the input weights and biases in hidden layers, and directly computing the output weights through a single learning process, which accelerates training significantly compared to traditional neural networks.

ELM excels in tasks where computational efficiency and scalability are paramount, making it particularly suitable for applications in gas recognition and other domains requiring rapid processing of high-dimensional data. However, ELM's lack of interpretability compared to conventional machine learning models remains a challenge. Future advancements in ELM are expected to focus on enhancing its interpretability, optimizing parameter selection, and addressing issues related to handling imbalanced datasets, further solidifying its role in practical applications requiring efficient and effective learning algorithms.

ELM is widely used in gas recognition for its fast learning speed and ability to handle large datasets. However, it lacks interpretability compared to traditional models, making it difficult to understand feature learning and predictions. It also faces challenges in parameter selection, which can affect performance and lead to overfitting, especially with imbalanced data.

#### Convolutional Neural Network

As a supervised deep learning method [97], CNN has shown considerable development prospects in the field of artificial intelligence. CNN is an improvement of the traditional neural network, with changes in the function and architecture of the layers. In general, CNN consists of five main layers [98]: the data input layer, convolution calculation layer, ReLU excitation layer, pooling layer, and fully connected layer. The convolutional layer utilizes multiple convolution kernels for feature processing, the pooling layer combines semantically similar features and prevents overfitting, and the final fully connected layer generates global semantic information for each neuron. Therefore, compared to other traditional neural network algorithms, CNN requires fewer parameters, reducing memory usage and improving efficiency. CNN’s ability to learn features and classification boundaries directly from the original input data makes it a suitable method for feature extraction in gas datasets. By using a self-designed ultra-low-power electronic nose system combined with CNN, Lee et al. [40] achieved a classification accuracy of 99.32% for five different gases. The electronic nose system can be driven by a battery and is expected to be used in environmental IoT applications.

Although several CNN models have been proposed for visual data processing, such as AlexNet [99], ResNet [100], and the inception series, these models are not directly applicable to gas classification. As a result, researchers have made efforts to modify CNN architectures for gas identification. Wei et al. [101] designed a network with two convolutional layers and two pooling layers to enhance the depth of learning. Experimental results demonstrated that the improved CNN model successfully identified CH_4_, CO, and their mixtures with an accuracy rate of 99.67%, surpassing SVM, MLP, and PNN. These studies collectively highlight the promising application prospects of deep learning methods in the field of gas identification.

CNN is widely used in gas recognition for its unique architecture, including convolutional, pooling, and fully connected layers. Convolutional layers extract local features from sensor data, pooling layers reduce dimensionality while preserving key information, and fully connected layers use these features for classification or regression. However, CNN faces challenges: it requires large labeled datasets for effective training and generalization, demands significant computational resources for handling high-dimensional data, and may need adjustments to adapt to dynamic gas environments.

#### Recurrent Neural Network

Recurrent neural network (RNN) is a type of FNN that differs in its connections between nodes, forming a directed graph along the time series. This unique structure allows RNN to effectively process input sequences of varying lengths, making it suitable for solving sequence-related problems like handwritten digit recognition. The hidden unit of RNN contains a state vector that retains historical information about past elements in the sequence [97]. This storage capability is facilitated by the weight between the hidden layers, which acts as the memory controller of the network, responsible for managing memory [102]. When RNN is time-expanded, it can be seen as a multi-layer FNN. However, it has been observed that learning long-term dependencies through gradient descent can be challenging. To address this problem, researchers have proposed using display memory to enhance the RNN, leading to the development of long-term and short-term memory networks [103]. RNN is particularly well-suited for processing time series data, and when combined with the characteristics of gas concentration time series, it can serve as a gas concentration prediction model. Song et al. [104] proposed a gas concentration prediction model based on RNN, which leveraged the characteristics of gas concentration time series to improve applicability and robustness. This model offered valuable insights and recommendations for coal mine safety management.

In gas recognition, RNN is frequently employed due to its capacity to model temporal dependencies in sensor data. Unlike feedforward neural networks, RNN possesses feedback connections that allow it to retain information about past inputs, making it suitable for sequential data processing tasks. RNN can effectively capture the dynamic nature of sensor responses over time in gas recognition, enabling accurate modeling and prediction of gas concentrations or types. However, RNN also faces challenges such as handling long-term dependencies, training stability issues, and computational complexity, particularly with large datasets.

### Ensemble Learning Method

Ensemble learning is a model optimization method that combines different algorithms [105] to improve the accuracy of the model [68]. It involves associating different models in parallel or series and integrating their results through averaging or voting. Therefore, the ensemble learning model is a common solution to the bottleneck of gas identification in deep learning applications. It overcomes the limitations of using a single model [106] and has the potential to enhance gas sensing performance. The classical BPNN is known to be slow in the training process and prone to falling into local minima. It effectively overcomes the drawbacks of BPNN. Moreover, the GA can enhance the prediction accuracy of the BPNN by optimizing the initial weight and threshold [93, 102]. Chu et al. [107] utilized the neural network algorithm to detect four different NO_2_ and CO mixtures and found that the GA-optimized BPNN algorithm exhibited superior performance in gas quantitative identification. Figure 6d–i illustrate the performance of BPNN and GA-BPNN in the quantitative detection of various CO and NO_2_ mixtures, respectively. Figure 6d–f describe the normalized confusion matrix of BPNN, the performance of quantitative detection of various mixtures of CO and NO, and the error between the predicted concentration and the actual concentration. Figure 6g–i describes the normalized confusion matrix of GA plus BPNN, the performance of quantitative detection of various mixtures of CO and NO, and the error between the predicted concentration and the actual concentration. These results indicate that GA effectively optimizes the parameters of BPNN. Therefore, combining the GA with other algorithms holds great potential in addressing the cross-sensitivity problem of multi-sensor arrays [108]. Furthermore, the generalization of ELM can mitigate the over-fitting phenomenon caused by a large number of hidden layers in BPNN [109]. For example, Wang et al. [110] proposed an E-nose system architecture based on edge computing, ensemble learning, and sensing clusters. Different ANN models were applied to each sensor unit to improve the system’s fault tolerance to sensor faults, and edge computing was used for data processing and analysis of E-nose. Based on this method, the fault-tolerant ability of the E-nose system for gas classification and concentration prediction is 10 and 18 times that of the traditional array sensors, respectively, which provides a new way for the development of highly fault-tolerant E-nose systems.

Ensemble learning methods can improve the classification performance, enhance the robustness and generalization ability of the system, deal with unbalanced data, and provide more interpretable model output in E-nose data processing, which is of great significance for improving the accuracy and reliability of odor recognition. For example, Xiong et al. [111] combined the gramian angular field (GAF) and CNN to preprocess the E-nose response data to classify the odor intensity levels of five gases. The core of the algorithm is to use GAF to convert one-dimensional time data into two-dimensional color images, and then design a classification model based on a multi-scale feature fusion network (MFFNet) for classification. Finally, MFFNet achieved the highest accuracy and macro average F1 score on the test set, which were 93.75% and 93.34%, respectively. This method extracts a unique fingerprint for each gas sensor signal, avoids complex feature engineering, and extends the traditional E-nose data processing ideas. Shi et al. [112] proposed a calculation method of gas feature attention mechanism (GFAM-Net) based on gas feature attention mechanism and lightweight CNN, aiming to effectively extract the key features of deep gas information and improve the detection performance of E-nose. At the same time, the effectiveness of the method is verified on different data sets based on E-nose. The experimental results show that GFAM-Net not only has fewer parameters and calculations but also has better performance in multi-learning models.

Sun et al. [113] proposed an adaptive convolution kernel channel attention (AKCA) module for gas feature extraction to identify the quality of soybeans from different producing areas. In comparison experiments with other attention mechanisms, AKCA-Net showed excellent performance, with an accuracy rate of 98.21%, an accuracy rate of 98.57%, and a recall rate of 98.60%. Wu et al. [114] observed that the transformer encoder (TE) and temporal convolute onal network (TCN) both showed excellent performance in processing sequence data, so they were combined to process E-nose data. Then the Bayesian parameter optimization algorithm is used to perform TE weighting on the data, and then important features are extracted. The classification accuracy is 99.8%. Zhang et al. [115] used six ZnO-based Micro-Electro-Mechanical Systems (MEMS) sensors to reduce the power consumption of the system to 36 mW. A particle swarm optimization algorithm is used for feature selection so that the feature is reduced from 72 to 5. Finally, these five features are input into the model combined with SVM and ANN to identify and quantify VOCs such as HCHO, C_2_H_5_OH, C_7_H_8_, and C_8_H_10_. The recognition accuracy and R^2^ reached 97.9% and 0.975%, respectively. Zhang et al. [116] proposed a fully connected temporal multi-layer graph convolutional network to predict gas concentration in gas sensor networks with spatio-temporal characteristics. The algorithm uses multiple graph convolution layers to capture the spatial features in the data, and then combines the gated recursive unit (GRU) to obtain the dynamic changes of the sensor network data and the time characteristics of the data. Finally, the fully connected layer is used to enhance the performance of the model.

Future research should prioritize the development of machine learning algorithms that can effectively adapt to and compensate for sensor drift over time, ensuring consistent performance of E-nose without frequent recalibration. Pan et al. [117] proposed a concentration prediction method for mixed gas recognition based on an attention-based hybrid domain adversarial learning transformer network (HATN-DA). Based on the label-free domain adaptation of Wasserstein distance, the label-free drift compensation and transfer calibration effects are realized: for public data sets, the drift compensation experimental accuracy of HATN-DA reaches 97.50%–100%; for the mixed gas dataset, the accuracy of HATN-DA reaches 98.79% in the batch transmission task, which is significantly improved compared with 90.16% (before transmission). Se et al. [118] used an online drift compensation framework based on active learning to achieve multi-task processing such as sensor drift, gas classification, concentration prediction, and labeling cost. Two strategies, including gas classification query strategy and concentration prediction query strategy, are used to capture drift information. At the same time, the self-renewal of online domain adaptive extreme learning machine is used to adapt to the changing sensor drift. This method can achieve the best generalization ability with the minimum labeling cost.

The ensemble learning method is widely utilized in gas recognition for its fast learning speed and capability to handle large-scale datasets. It employs a single hidden layer of randomly generated neurons, followed by a linear output layer, making it computationally efficient. However, the ensemble learning method may lack interpretability compared to traditional machine learning methods.

## Applications and Challenges

### Food Quality and Safety

With the increasing complexity of the food supply chain, ensuring food safety and quality has become increasingly important throughout the entire process from farm to fork. Traditionally, there have been several conventional methods used to assess food quality, such as polymerase chain reaction (PCR), enzyme-linked immunosorbent assay, back-flow immune bands, gas chromatography, high-performance liquid chromatography, and mass spectroscopy [119]. While these methods offer reliable technology, they are often time-consuming and require significant labor.

In contrast, pattern recognition methods based on multi-sensor arrays not only allow for rapid non-destructive testing but also enable the tracing of product origins, identification of authenticity, and early detection of pests and diseases [120], which is expected to become a non-destructive quality assessment tool that can replace traditional methods. This section focuses on the application and challenges of pattern recognition methods in assessing the quality of fruits, vegetables, fish, and beverages. Table 3 provides a comprehensive overview of how pattern recognition algorithms are applied to address cross-sensitivity challenges in the domain of food quality and safety. It details the algorithms employed, the specific gases they relate to, and the count of gas sensor arrays involved.

**Table 3:** Summarizes and describes the different pattern recognition methods applied to the quality assessment of food quality and safety

Species	Purpose	Detectable gases	Sensor Number	Pattern Recognition	References
Fruits and vegetables	Detection and classification of early infected citrus fruits	Organic solvent, H_2_, VOCs, CH_4_, sulfur organism, sulfur chlorinate, methane aliphatics	8	PCA, LDA	[124]
	Monitoring the shelf life of tomatoes	C_7_H_8_, NO_2_, C_6_H_6_, H_2_, CH_3_CH_2_CH_3_, CH_3_, H_2_S, CO, NH_3_, CH_4_	10	PCA, LDA	[272]
	Tracking the growth of peach fungi	C_7_H_8_, NO_2_, C_6_H_6_, H_2_, CH_3_CH_2_CH_3_, CH_4_, H_2_S, CO	10	PCA, PLSR	[273]
	Evaluation of Strawberry Freshness with Different Polymer Packaging	CH_4_, C_2_H_5_OH, CH_3_CH_2_CH_3_, C_4_H_8_, H_2_, natural gas, organic solvent vapor, H_2_S (gas), alcohol, ammonia-benzene-carbon dioxide (CO_2_)	8	PCA, LDA, SVM	[274]
	Distinguish strawberry juice with different processing methods	C_7_H_8_, NO_2_, C_6_H_6_, H_2_, CH_3_CH_2_CH_3_, CH_3_, H_2_S, CO, alcohol’s, aromatic compounds	10	ELM, LVQ, Lib-SVM	[275]
	Classification of cumin, parsley, and other seeds	CH_4_, CH_3_CH_2_CH_3_, C_4_H_10_, H_2_, natural gas, vapors of organic solvents, H_2_S, alcohol, NH_3_, C_6_H_6_, smoke, CO_2_	8	PCA, 2D-LDA, U-PLS-DA, PARAFAC-LDA	[276]
	Traceability of soybean quality	Aromatic compounds, NH_3_, H_2_, alkanes, CH_4_, ethanol, polar compounds, NO_x_, sulfur compounds, and many terpenes	10	LRCNN	[130]
	Detection of apple fungal infection	C_7_H_8_, C_6_H_6_, CH_3_CH_2_CH_3_, CH_4_, CO, NO_2_, H_2_, H_2_S	10	K-NN RF, SVM, CNN, BPNN, PSO-BPNN, GWO-BPNN, SSA-BPNN	[126]
	Distinguish between fresh and moldy apples	C_6_H_5_CH_3_, NO_2_, C_6_H_6_, H_2_, C_3_H_8_, CH_4_, H_2_S, C_2_H_5_OH	10	LDA, BPNN, SVM, RBFNN	[277]
	Classification of fresh fruit and spoiled fruit	H_2_S, C_2_H_5_OH, C_3_H_6_O, H_2_, CO, NH_3_, VOCs	5	MLP, PCA, ReliefF	[278]
	Detection of adulteration of cherry tomato juice	Aromatics, NO_x_, NH_3_, aromatic molecules, H_2_, CH_4_, CH_3_CH_2_CH_3_, aliphatic non-polar molecules, sulfur-containing organics, broad alcohols, aliphatics	10	PCA, CDA, LVQ, SVM	[279]
	Berry maturity assessment	C_2_H_5_OH, LPG, CH_4_, Coal gas, CO, combustible gas, steam organic solvents, C_4_H_10_, CH_4_, C_3_H_8_, sulfide, H_2_S, NH_3_, C_7_H_8_	10	PCA, LDA, ANN	[132]
Fish and meat	Freshness evaluation of horse mackerel	aroma component, aromatic components, short-chain alkanes, inorganic sulfide, alcohols, organic sulfides, NH_4_OH, H_2_, NO_x_, alkane, long-chain alkanes	10	ANN, RFR, XGBoost, SVM	[142]
	Freshness classification	H_2_S, NH_3_, C_2_H_5_OH, C_2_H_4_	4	CNN	[122]
	Determination of fish quality parameters	CH_4_, CO, C_2_H_5_OH, C_4_H_10_, CH_3_CH_2_CH_3_, H_2_, CO, NH_3_, C_6_H_6_, CO_2_	6	ANOVA	[280]
	Meat and fish quality assessment	C_2_H_5_OH, CH_4_, CO, H_2_, NO_2_, O_3_, NO_x_, VOCs	4	PCA, K-NN, PLSR-DA	[281]
	Detection of fish freshness	O_3_, NO_x_, C_2_H_5_OH, smoke, VOCs, CO, NH_3_	8	ANN, SVM, KNN	[282]
	Discrimination and prediction of multiple beef freshness	Aromatics, NO_x_, NH_3_, aromatic molecules, H_2_, CH_4_, CH_3_CH_2_CH_3_, aliphatic non-polar molecules, sulfur-containing organics, broad alcohols	10	MD, PCA, LDA, BPNN, CRNN	[283]
	Identification of pork adulteration in lamb minced meat	Aromatics, NO_x_, NH_3_, aromatic molecules, H_2_, CH_4_, CH_3_CH_2_CH_3_, aliphatic non-polar molecules, sulfur-containing organics, broad alcohols, aliphatic, chlorine-containing organics	10	CDA, BDA, PLS, MLR, BPNN	[284]
	Beef Quality Classification	H_2_S, RCHO, ketones, CH_4_, liquefied petroleum gas, C_6_H_6_, smoke, NO_x_, (LPG), CH_3_CH_2_CH_3_, i-butane, NH_3_, CO_2_, C_2_H_5_OH, C_6_H_14_, CO	11	DWTLSTM, LSTM, K-NN, LDA, MLP, SVM	[285]
Beverage category	Pure juice and industrial juice classification	C_4_H_10_, CH_4_, CO, NH_3_, C_6_H_6_, H_2_, C_2_H_5_OH, SO_2_, C_3_H_8_, steam organic solvents	9	ANN, PCR	[286]
	Detection of detergent powder in raw milk	H_2_, C_2_H_5_OH, LPG, cooking fumes, H_2_S, air, CH_4_, CH_3_CH_2_CH_3_, C_4_H_10_, organic solvent vapors, C_7_H_8_, C_8_H_10_, VOCs	8	PCA, SVM, ANFIS, MANOVA, LDA,	[160]
	Identification of milk adulteration	VOCs, H_2_S, C_2_H_5_OH, C_7_H_8_, C_8_H_10_, organic solvent vapours, alcohol, cooking fumes, CH_4_, C_4_H_10_, CH_3_CH_2_CH_3_,	8	PCA, LDA, SVM	[23]
	Identify the source of milk	Polluting gas, volatile substances of alcohol and organic solvents, CH_4_, NH_3_, C_6_H_6_, freon gas, C_2_H_5_OH, CO, VOCs	7	PCA, LDA, LR, SVM, RF	[287]
Miscellaneous	Detection of edible oil oxidation	SO_2_,CO,combustible gas, CH_4_, C_3_H_8_, C_4_H_10_, NH_3_, C_2_H_5_OH, steam organic solvents, C_6_H_6_, sulfide, H_2_S	8	CA, PCA, PLS, QDA, SVM	[288]
	Identification of edible oil storage period	H_2_, CO, CH_4_, C_2_H_5_OH, C_4_H_10_, CH_3_COCH_3_, CH_2_O	4	K-NN, LDA, SVM	[289]
	Classified protein, egg yolk, and whole egg	C_4_H_10_, NO_2_, C_6_H_6_, H_2_, CH_3_CH_2_CH_3_, CH_3_, H_2_S, CO	10	PCA, LDA, KNN, SVM	[290]
	Essential oil classification	CO, combustible gases, urban gases, CH_4_, NH_3_, C_6_H_6_, sulfides, H_2_, alcohols, organic solvents, SO_2_, C_3_H_8_, C_4_H_10_	9	PCA, LDA, SVM	[291]
	Tea quality classification	NH_3_, H_2_S, C_2_H_5_OH, C_7_H_8_, C_8_H_10_	4	PCA, FCM, RBF, PNN, MLP	[292]
	Monitoring of rice storage aging process	H_2_, natural, organic solvent vapours, C_2_H_5_OH, H_2_S, CH_4_, CH_3_CH_2_CH_3_, C_4_H_10_	7	BP, RBF, LVQ	[293]
	Rice quality identification	Alkenes, broad-methane, sulfur-containing organics, aromatics, NO_x_, NH_3_, aroma constituent, H_2_, alcohols, sulfur organic compounds, CH_4_, aliphatic	10	IACCN	[294]

#### Fruits and Vegetables

It has been proved that gas sensors can be used for disease prediction and safety assessment of fruits and vegetables. The detection of aroma and VOCs is an important method to evaluate plant infection. Taking effective measures to prevent crop infection can improve the quality and yield of fruit and vegetable crops. In addition, during the processing, transportation, and storage of fruits and vegetables, some VOCs are released from these perishable foods due to the breakdown of nutrients, which can be used as the evaluation criteria for their freshness.

The effectiveness and classification accuracy of gas sensors in food freshness monitoring can be significantly improved through pattern recognition methods. For instance, Chen et al. [121] conducted a study using a MOS-based sensor array to evaluate the freshness of broccoli samples during storage. They employed a PCA model to differentiate between fresh, medium fresh, and spoiled broccoli samples, achieving a classification rate of 95.37%. The canonical discriminant analysis (CDA) model successfully distinguished broccoli samples based on their freshness with 100% accuracy. Similarly, Ren et al. [122] proposed an E-nose system based on CNN that effectively classifies the freshness of various types of food. Through the analysis performed by CNN, the system achieved a final freshness classification accuracy of 97.3% for 20 different types of foods.

**Fig. 7:**
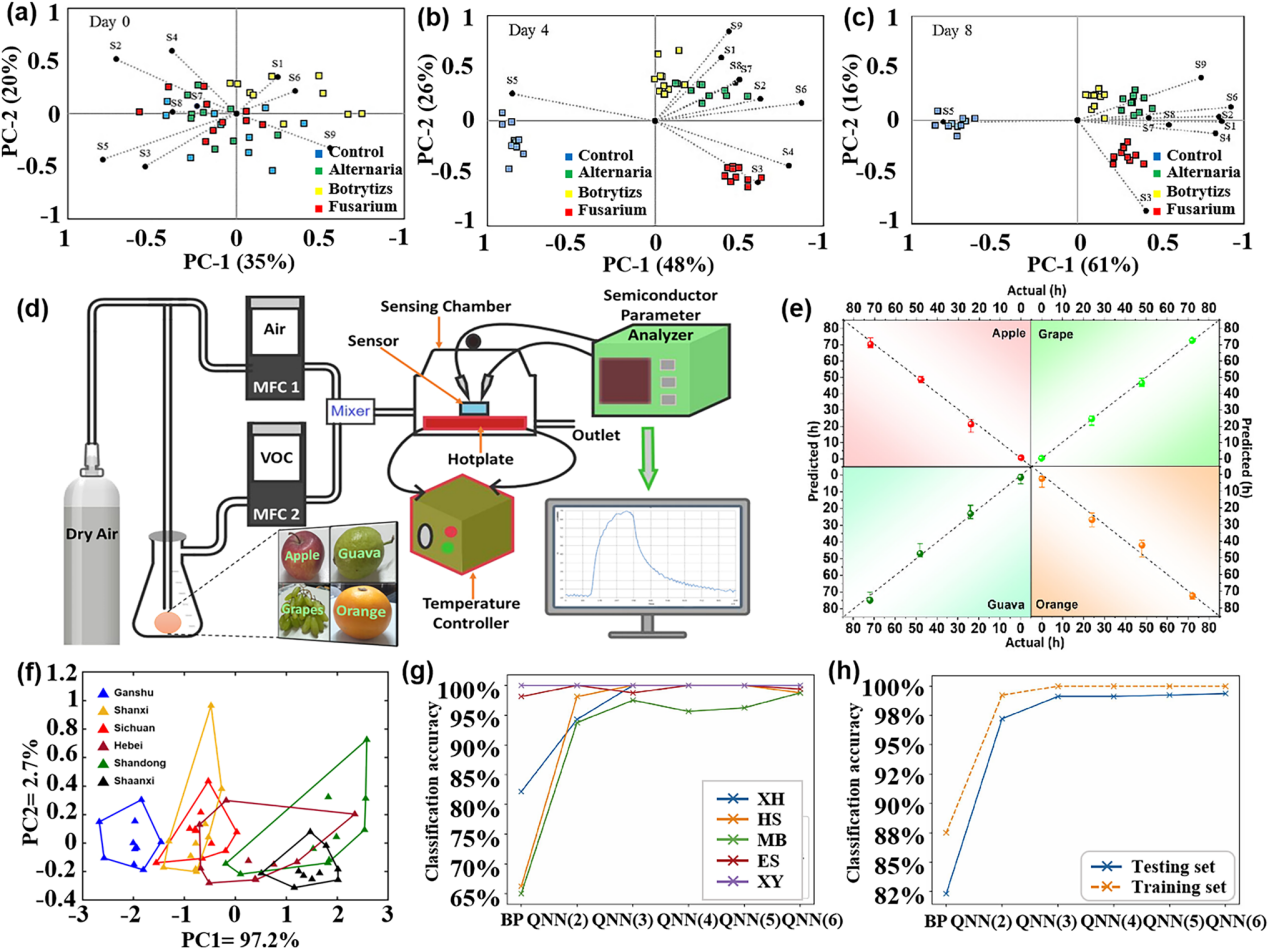
Pattern recognition for fruits and vegetables. **a** PCA double images of garlic treated with different infections on day 0. **b** PCA double images of garlic treated with different infections on day 4. **c** PCA double images of garlic treated with different infections on day 8. Reproduced with the permission from Ref. [125], Copyright Elsevier 2022. **d** VOCs of four fruits were collected at different time periods. **e** The neural network model was used to quantify the storage time of four fruits. Reproduced with the permission from Ref. [128], Copyright American Chemical Society 2023. **f** PCA results corresponding to three sensor data. Reproduced with the permission from Ref. [129], Copyright Wiley 2021. **g** Classification accuracy of each green tea using QNNs with different numbers of quantum intervals and BPNN. (XH represents Xihu Longjing, HS represents Huangshan Maofeng, MB represents Mabian Lucha, ES represents Enshi Yulu, and XY represents Xinyang Maojian). **h** Average classification accuracy obtained by corresponding BPNN and QNN under different quantum intervals. Reproduced with the permission from Ref. [131], Copyright Elsevier 2023

In order to detect agricultural product corruption at an early stage, multi-sensor arrays combined with pattern recognition methods, are designed to predict product corruption quickly and reduce the deterioration rate. To improve the reliability of the results, researchers have considered using data fusion methods to detect samples. Rutolo [123] developed a low-cost gas sensor array that monitors soft rot in potatoes by analyzing the odor released by infected samples. The PCA model achieved 100% accuracy in distinguishing healthy and infected potato samples. This method provides a simpler and more reliable approach for sensor monitoring systems that traditionally rely on human skills. Wen et al. [124] independently developed a sweeping E-nose system to detect the presence of early infestation by Bactrocera dorsalis in citrus fruits. The results showed that LDA could successfully distinguish different types of treatments with a success rate of 98.21%. This system holds promise for on-site detection of postharvest pests of citrus fruits under market conditions. Makarichian et al. [125] concerned that there was little literature on the pattern of volatiles in infected garlic bulbs, so they used 9 MOS sensors (in Fig. 7a–c, s1–s9, respectively, represent MQ-2, MQ-3, MQ-4, MQ-5, MQ-6, MQ-7, MQ-8, MQ-9, and MQ-135) for experiments. Since each sensor has a different detection range, it is impossible to distinguish whether garlic is infected based on the output of the sensor array alone. After normalizing the sensor data, Fig. 7a–c are PCA double graphs, which show the discrimination of garlic samples after different infection treatments on the 0th day, the 4th day and the 8th day. Although it could not be distinguished on day 0, the inoculated samples were distinguished from the CO samples on day 4, indicating that the garlic infection could be identified according to the aroma characteristics in the early stage. Zhao et al. [126] used eight different algorithms, including KNN, RF, SVM, CNN, BPNN, particle swarm optimization-back-propagation neural network (PSO-BPNN), gray wolf optimization-back-propagation neural network (GWO-BPNN), and a sparrow search algorithm-backward propagation neural network (SSA-BPNN) models, to analyze the odor information of apples collected by an E-nose and distinguish between infected and non-infected apples. GWO-BPNN combines the grey wolf optimization (GWO) algorithm with BPNN. The GWO algorithm simulates the social behavior of a grey wolf pack, optimizing the performance of the neural network by adjusting its weights and biases. SSA-BPNN integrates the sine cosine algorithm (SSA) with BPNN. The SSA algorithm mimics the variation process of the sine cosine function, enhancing the convergence speed and generalization ability of the neural network.

However, it has been pointed out that the use of E-nose for detecting apple quality can be easily interfered with by external factors. Du et al. [127] combined E-nose and CNN technology to achieve rapid detection of the quality changes of sauerkraut. The model they trained only after 50 cycles, the accuracy of the sample test set reached 93%, indicating that CNN has a high performance in predicting the quality of sauerkraut. Mahata et al. [128] developed a solid-state chemical resistance-based sensing system that can monitor fruit freshness in real-time. Apples, guava, oranges, and grapes were selected as monitoring objects, respectively. The specific sensing process is shown in Fig. 7d. The neural network was used to analyze the VOCs collected in different periods. Figure 7e shows the quantitative results of the model for different fruits, and each quadrant represents a fruit. The average errors of the model for the four fruits were 7.32%, 5.25%, 8.94%, and 9.71%, respectively. It is obvious that the freshness of fruit samples can be predicted by monitoring the storage time of fruits.

In addition, the appropriate pattern recognition method can also be used to determine the source of fruits and vegetables and achieve quick anti-counterfeiting traceability of products. Mao et al. [129] based on the two eigenvalues obtained after filtering, combined with 22 model analysis methods to trace the source of 6 kinds of Chinese red pepper. Firstly, principal component analysis—discriminant index is used to obtain the optimized eigenvalues corresponding to the three sensors, and then PCA analysis is performed on the eigenvalues of this group, as shown in Fig. 7f. It can be seen that there are 5 kinds of red pepper cross phenomenon, so the authors used 10 times cross varicose veins and minimum classification error to extract the filter characteristic value. Finally, based on these two eigenvalues, the ensemble learning algorithm shows more than 90% resolution. Considering the sensor’s cross-sensitivity, Lin et al. [130] proposed a long-range convolutional neural network (LRCNN) algorithm to achieve soybean quality traceability, which effectively reduces the number of parameters and prevents the degradation of deep CNN. The classification accuracy of the algorithm reaches 98.37%. Similarly, the E-nose combined with the pattern recognition method is used to achieve the purpose of soybean traceability. Fu et al. [131] used the method of E-nose combined with a quantum neural network to identify five different geographical locations of green tea. Their method is similar to the traditional BPNN, but its transfer function is formed by the superposition of quantum intervals formed by multiple sigmoids and can be adjusted during training. In the experiment, they trained each quantum neural network (QNN) and BP neural network with different initial quantum intervals. It can be seen from Fig. 7g that the performance of all QNNs is better than that of BPNN for the classification accuracy of each green tea, and the classification accuracy increases with the increase of the number of quantum intervals (Fig. 7h). In order to understand the recognition results in more detail, the researchers also provided the confusion matrix of BPNN and QNN under two quantum intervals. The results showed that the accuracy of BPNN and QNN with 2 quantum intervals (QNN (2)) in identifying Xinyang Maojian reached 100%. However, compared with BPNN, QNN (2) has fewer false predictions, especially in identifying green tea in other regions, especially for Huangshan Maofeng and Mabian Lucha. This shows that QNN (2) has better performance in the identification of green tea producing areas. The AKCA-Net proposed by Sun et al. [113] also shows excellent performance, achieving an accuracy of 98.21%, precision of 98.57%, and recall of 98.60%. AKCA-Net utilizes the adaptive k-clone algorithm (AKCA) to optimize the structure and parameters of the neural network. The AKCA combines the features of clone selection and adaptive K-value. This combination enables adaptive selection and adjustment of the neural network's topology and parameters. As a result, the AKCA enhances the network's performance and generalization ability in tasks such as gas recognition. Exploring techniques for efficient data dimensionality reduction and sensor data fusion can enhance the performance of E-nose.

Maturity plays a crucial role in assessing the quality of fruits and vegetables as it impacts their taste and storage characteristics. To determine the maturity stages of these products, an E-nose can be employed to analyze the VOCs released by them. For instance, Aghilinategh et al. [132] utilized an E-nose to detect the five ripeness grades of berries, namely whiteberry and blackberry. The researchers employed three different algorithms for pattern recognition, LDA can distinguish blackberry well and the accuracy rate is 96.67%. ANN achieved 100% accuracy in the blackberry classification and 88.3% accuracy in the blackberry classification.

#### Fish and Meat

In addition to fruit and vegetable products, fish and meat products are important sources of protein for nutritional intake. It is crucial to detect the freshness, shelf life, and early deterioration of these foods during transportation and storage to ensure their safety and freshness. One typical sign of fish or meat spoilage is the emission of VOCs, such as ammonia [133]. Ma et al. [134] observed that the gas sensor response to beef, pork, fish, and chicken stored at 30 °C for 20 h varied, indicating different levels of deterioration. Therefore, monitoring the fish quality has also become an important application area for multi-sensor array gas detection [135]. This includes detecting the freshness of different types of meat under various storage time, identifying meat adulteration [136], determining meat species identification [137], and assessing the authenticity of meat products [138, 139].

**Fig. 8:**
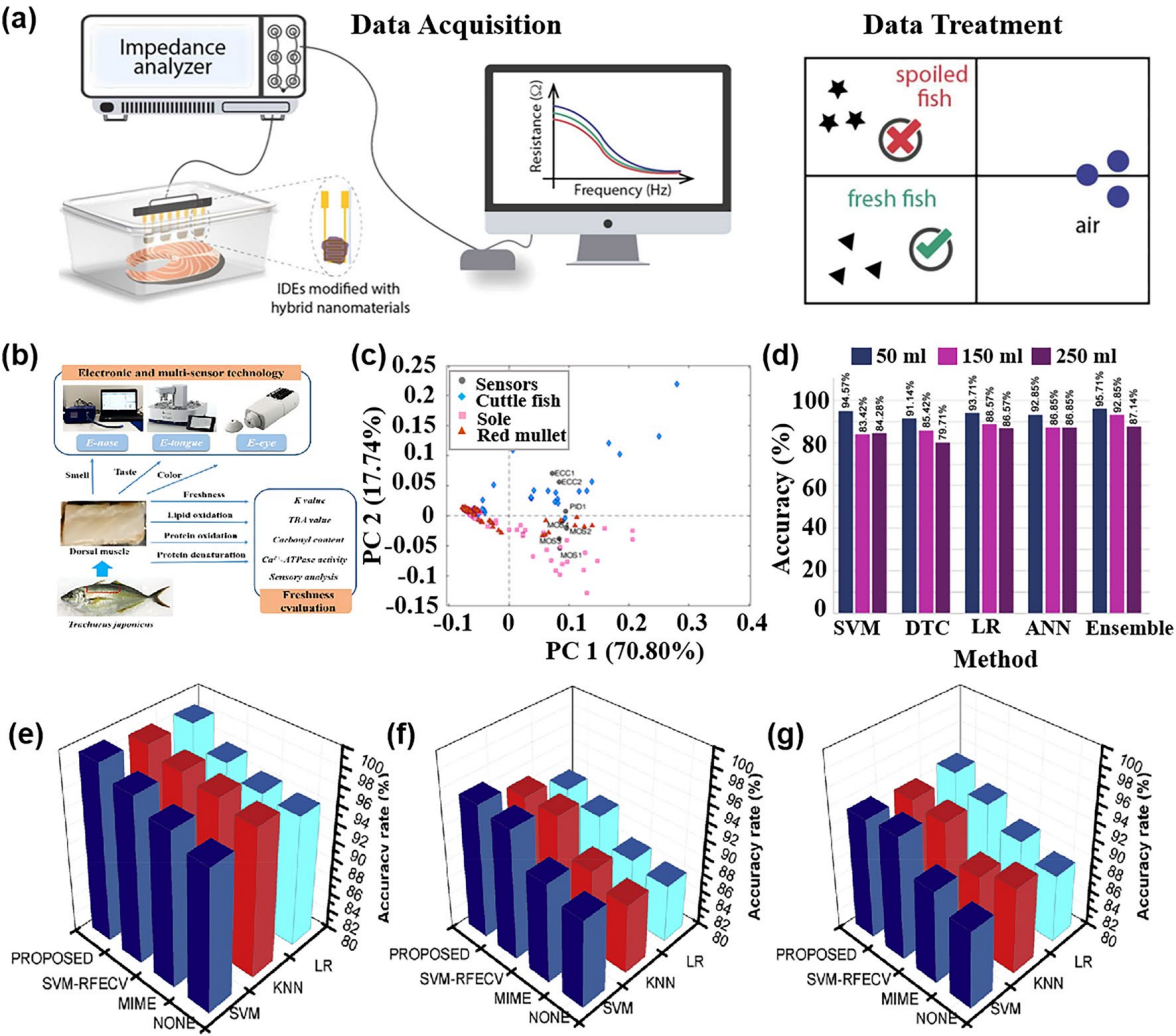
Pattern recognition methods in fish quality detection. **a** An E-nose system for monitoring fish spoilage. Reproduced with the permission from Ref. [141], Copyright Elsevier 2022. **b** E-nose, E-tongue, and colorimeter. Reproduced with the permission from Ref. [142], Copyright Elsevier 2023. **c** PCA double graph of the electronic nose data of the three fish species, the color difference represents the different freshness levels determined according to the K-means. (PID stands for photoionization detector, and ECCs stands for electrochemical cells). Reproduced with the permission from Ref. [143], Copyright Elsevier 2022. **d** Comparison of classification accuracy obtained by different classification methods under three gas chambers. (LR stands for logistic regression, and DTC stands for decision tree classifier). Reproduced with the permission from Ref. [105], Copyright Elsevier 2022. **e**‒**g** Comparison of classification accuracy for three grades of ham using four different prediction models: MIME (Mutual Information Mixed Evaluation), SVM-BFECV (Support Vector Machine-Backward Feature Elimination with Cross-Validation), MIME-SVM-RFECV, and original features. Reproduced with the permission from Ref. [147], Copyright Elsevier 2021

The nutritional value and delicious taste of fish meat make it highly popular among people. The economic value of fish meat is greatly influenced by its freshness. Therefore, there is a need to develop a fast, sensitive, and cost-effective tool for reliable freshness detection and shelf life prediction. In a similar experiment, Karunathilaka et al. [140] employed MOS sensors combined with SVM modeling to classify mahi-mahi, croaker, red snapper, and weakfish. The correct classification rates were 100%, 100%, 97%, and 97% respectively. Andre et al. [141] prepared SiO_2_:In_2_O_3_, SiO_2_:ZnO nanofibers by electrospinning and used them as a sensing platform (Fig. 8a). The discriminant analysis method was used to identify volatile amines emitted during meat spoilage to detect the degree of spoilage of fish. Li et al. [142] conducted an experiment using an E-nose, E-tongue, and colorimeter in combination with four different machine learning algorithms to quantitatively evaluate and predict the freshness of horse mackerel (Trachurus japonicus), as depicted in Fig. 8b. In the experiment, ANN, extreme gradient boosting (XGBoost), random forest regression (RFR), and support vector regression were employed to establish a freshness prediction model for fish meat. The results indicated that the ANN, XGBoost, and RFR models achieved the best outcomes for protein oxidation degree, lipid oxidation degree, and protein denaturation degree, respectively. Hence, these three indicators demonstrate that the utilization of gas sensors combined with machine learning algorithms can effectively evaluate and predict the freshness of frozen fish. Grassi et al. [143] designed a convenient and cheap E-nose system consisting of four MOS sensors to achieve the freshness of different seafood species only through a single classification model. Figure 8c is a PCA double image of the electronic nose data of the three fish species. The color difference represents the different freshness levels determined by the K-means. It can be seen that the classification of samples along PC1 is only related to the number of storage days. This result is consistent with the PCA double graph results of a single sample, which lays a foundation for the design of a single classification model.

Meat is a perishable product. During storage, changes in chemical composition result in the emission of many volatile compounds [144]. In the case of meat adulteration, Tian et al. [145] employed an E-nose system with 10 selective MOS gas sensors to detect adulteration in minced mutton mixed with pork. Various pattern recognition methods, including normative discriminant analysis, PCA, stepwise LDA, multiple linear regression (MLR), partial least squares (PLS), and BPNN, were used for analysis. Among these, canonical discriminant analysis achieved excellent clustering of pure and adulterated samples, with an accuracy rate of 93.10%, effectively distinguishing mutton and pork adulteration. Similarly, Wang et al. [136] successfully identified adulteration in mutton and duck meat using an E-nose system and employed solid-phase microextraction for volatile compound extraction. The sensor data was analyzed using linear regression, fisher LDA (FLDA), and multilayer perceptron neural networks analysis, with an accuracy exceeding 95%. Wakhid et al. [105] studied the effect of input gas concentration on meat adulteration classification using E-nose. In the experiment, seven kinds of mixed beef and pork were selected as samples, and different concentrations of gas were obtained using 50, 150, and 250 mL gas chambers, respectively. Finally, the different combinations of statistical methods and four classification methods are compared to find the best method. Figure 8d is a comparison of the classification accuracy of different classifiers under three gas chambers. It can be seen that no matter which classification method, the greater the gas concentration, the better the classification effect. Similarly, ensuring the quality of meat products is crucial for food safety supervision. In a study by Putri et al. [146], an E-nose based on supervised machine learning (LDA, quadratic discriminant analysis (QDA), K-NN, and RF) was used to classify meat floss derived from beef, chicken, and pork. The LDA model with five windows achieved an accuracy of over 99% in distinguishing the three types of meat floss, and the analysis results of the E-nose were validated by spectral data. These experiments highlight the effectiveness of pattern recognition methods as a powerful analytical tool for identifying meat adulteration and evaluating freshness using pattern recognition method.

Qian et al. [147] proposed a hybrid feature optimization method, which uses the mutual information hybrid evaluation method to filter out irrelevant features, and then uses the packaging method based on support vector machine backward feature elimination cross-validation (SVM-BFECV) to remove multi-collinear features. The E-nose system combined with this method has achieved good results in the classification of three grades of ham. As shown in Fig. 8e–g, the prediction accuracy of this method is compared with the other three models (mutual information mixed evaluation (MIME), SVM-BFECV, and PROPOSED, which represents MIME-(SVM-RFECV)) in three grades of ham. Obviously, the MIME-(SVM-BFECV) method performs best. Choosing a good feature optimization method is also the key to the generalization of E-nose applications in the future.

#### Beverage

The early use of pattern recognition methods in analyzing gas sensor responses has been successfully applied to various aspects of beverage analysis. For example, it has been used for detecting beverage adulteration [148], quality assessment [149], and variety classification [150]. This section mainly introduces the application of pattern recognition methods in the detection of juice, wine, and milk quality by gas sensor array.

Quality inspection of fruit juice is of multiple importance, including food safety assurance and product quality control. Through quality testing to ensure the safety of fruit juice, including the detection of harmful microorganisms, heavy metals, pesticide residues, and other substances harmful to human health, to ensure the health and safety of consumers. In addition, quality inspection helps to control the quality of fruit juice, including taste, color, smell, nutrients, etc., to ensure the consistency and stability of the product, improving consumer satisfaction and trust.

**Fig. 9:**
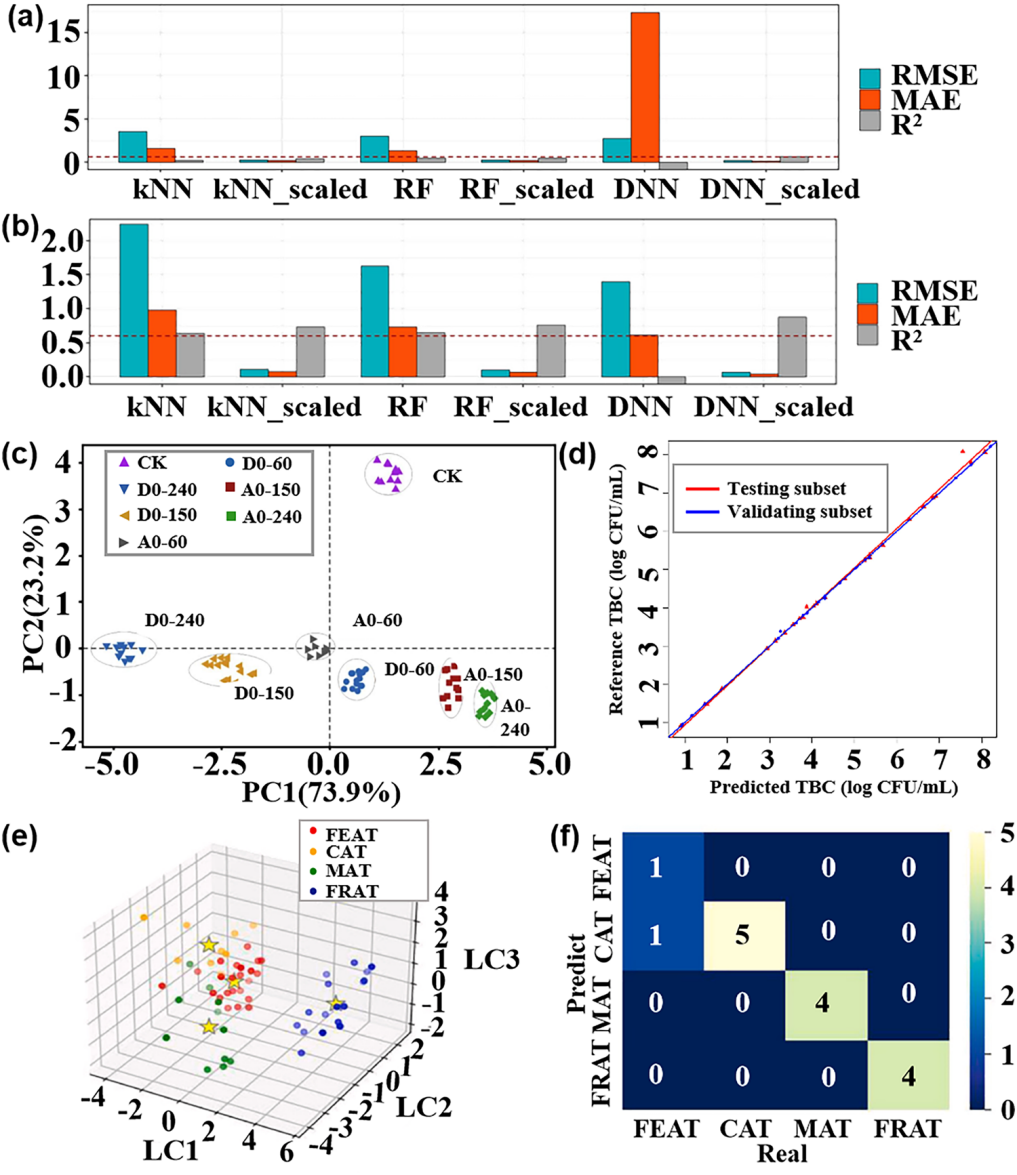
Pattern recognition methods for different drinks. **a** Prediction performance of each model before data interpolation. **b** Prediction performance of each model after data interpolation. Reproduced with the permission from Ref. [152], Copyright Elsevier 2023. **c** PCA diagram of different apple wines. (D0-60, D0-150, and D0-240 refer to ciders with 60, 150, and 240 mg N/L of DAP added, respectively. A0-60, A0-150, and A0-240 indicate ciders supplemented with 60, 150, and 240 mg N/L of the amino acids mixture, respectively). Reproduced with the permission from Ref. [155], Copyright Elsevier 2022. **d** Relationship between the predicted value and the reference value of TBC in the test set and the validation set. Reproduced with the permission from Ref. [162], Copyright Elsevier 2021. **e** Visualization of LDA model classification shows that the four categories are well distinguished. **f** Confusion matrix of four categories on the test data set. Reproduced with the permission from Ref. [163], Copyright Elsevier 2023

Qiu et al. [82] employed an E-nose to detect two food additives, namely benzoic acid and chitosan. To mitigate the risk of multicollinearity, SVM, RF, ELM, and PLSR were employed to establish regression models for the E-nose signal and food additive content in juice. The regression accuracy of different models was compared, revealing that the monitoring model based on ELM and RF yielded the best results. Furthermore, LDA was employed for qualitative analysis, demonstrating that the signal provided by the E-nose effectively characterized the sample. Yang et al. [151] used the ANN model to evaluate the relationship between market purchases of juice and homemade juice and human sensory pleasure. By fitting the sensory attributes of human beings, it provides scientific support for the evaluation of juice based on multi-sensory technology, which is helpful for the improvement and optimization of fruit products. Ren et al. [152] predicted the formula and process of vegetable and fruit beverages by combining electronic sensing technology with machine learning. Data interpolation technology is used to process the data. Figure 9a shows the performance of each model before data interpolation, and Fig. 9b shows the performance of each model after data interpolation. The results show that after data interpolation, root mean square error and mean absolute error decrease, and R^2^ increases significantly, which indicates that the change of process conditions will affect the prediction performance of the model.

For alcoholic drinks, due to its special production process, the concentration change of the compound affects its quality to a certain extent. Quality inspection can help prevent fake and shoddy products in the wine market, and ensure the authenticity and traceability of products by detecting the composition and quality characteristics of wine.

Stevan et al. [153] used a low-cost E-nose to classify blueberry wine samples of four different species harvested in different periods and used six classifiers to analyze the wine aroma data set. The six classification tools obtained excellent accuracy values (greater than 99.7%), and the data separability of the three tools reached 100%. From a computational point of view, K-NN and ANN are easy-to-implement tools with a low computational workload structure and are suitable candidates for future embedded system applications. These systems allow the online classification of samples. Similarly, in the study of wine quality, Rodriguez Gamboa et al. [154] focused more on the threshold of acetic acid in wine, and its content is very important for wine quality control. It is worth mentioning that to obtain fast online detection, the authors use the deep MLP classifier to process the original data. The classification of the degree of corruption of the three wines was achieved within 3 s of gas injection. This method is 63 times faster than the traditional SVM classifier method. Xu et al. [155] analyzed the aroma characteristics of cider using an E-nose, as shown in Fig. 9c, which is a PCA analysis of the E-nose in response to different cider. It can be seen from the figure that both PC1 and PC2 can distinguish the apple wine samples well, and it also proves that the presence of inorganic nitrogen and organic nitrogen will affect the aroma of apple wine.

In addition to fruit juice drinks or wine, dairy products also have a complex composition, containing over 100 compounds. These compounds include low-grade fatty acids, acetone, acetaldehyde, carbon dioxide, and other volatile substances that can affect the smell of milk [156]. Proteins, fats, and lactose in milk undergo a decomposition process that produces numerous volatile compounds [157, 158]. Therefore, pattern recognition based on sensor arrays is widely employed in the detection of milk and dairy products. This technology can detect various adulterations, such as the addition of skim milk and reconstituted milk powder [159], detergents in raw milk [160], cheese adulteration, and margarine adulteration [161]. In order to achieve rapid prediction of the total number of bacteria (TBC) in milk, Yang et al. [162] proposed a combination of E-nose and ANN to achieve rapid assessment of milk microbial quality. Through experiments, they found that the correlation between the predicted value and the reference value of TBC using the ANN model is as high as 0.99 (Fig. 9d), and it is surprising that the measurement of the single product only takes about 2 min. This result greatly indicates that the combination of E-nose and ANN can quickly and sensitively predict the TBC value in the milk. In order to make up for the gap in the establishment of a rapid identification method for yogurt aroma types, Hong et al. [163] combined E-nose data with a variety of machine-learning methods to establish an aroma classification model. Figure 9e is an LDA model of four aromas, showing good dispersion. In addition, the LDA classification model shows more than 90% classification accuracy on the test set and the training set. Figure 9f is the confusion matrix of the four classifications in the test data set. Only the fermented aroma type is misclassified, and the others are correctly classified.

### Environmental Monitoring

Due to the increase in human activities, air pollution has become a major threat to human health and safety [164]. Studies have shown that exposure to air pollutants such as particulate matter and ozone is associated with higher rates of mortality and hospitalization due to respiratory and cardiovascular diseases [165]. In addition, long-term exposure to contaminated environments can promote the spread of the virus [166]. Therefore, it is of great significance to monitor air quality in real-time, ensure industrial safety, and protect the living environment. This section will discuss the application of a multi-sensor array combined with a pattern recognition method in environmental monitoring, focusing on the above three aspects. Table 4 presents a summary of how pattern recognition algorithms are utilized to tackle cross-sensitivity issues within the realm of environmental protection. It includes information on the algorithms used, the gases they are associated with, and the total number of gas sensor arrays deployed.

**Table 4:** Summarizes and describes the different pattern recognition methods used in environmental monitoring

Species	Purpose	Detectable gases	Sensor Number	Pattern Recognition	References
Flammable and explosive toxic gas	Industrial harmful gas process monitoring	CH_3_COCH_3_, C_2_H_5_OH, C_6_H_14_, C_8_H_10_, isopropamide vapors	5	PCA, MVLR	[295]
	Toxic gas detection, identification, concentration estimation	CH_4_, LPG, H_2_, CO, C_2_H_5_OH, smoke	2	Levenberg–Marquardt, ANN, LSR	[179]
	Qualitative and quantitative detection of flammable liquid	H_2_, CO, H_2_S, VOCs, C_2_H_5_OH, CH_4_, C_4_H_10_, Stench, etrahydrofuran, turpentine, petroleum, gasoline, natural gas, lacquer thinner, tetrahydrofuran, CH_3_CH_2_CH_3_, lacquer thinner	14	BPNN, PCA	[296]
Outdoor harmful gas	Detection and classification of volatile gases in diesel–biodiesel blends	C_2_H_5_OH, CO_2_, combustion gases, NH_3_, C_6_H_6_, sulfide steams, SO_2_, H_2_S, CH_4_, CH_3_CH_2_CH_3_, organic solvents steam, C_7_H_8_	8	SVM, QDA, LDA	[297]
	Five typical odors identification	H_2_, CO, combustible gas, C_2_H_5_OH, C_7_H_8_, CH_4_S, C_6_H_6_, C_4_H_10_, CH_4_, NH_4_	9	RF, BPNN, PCA, LDA, SVM	[298]
	Urban road NO_x_ monitoring	CO_2_, HCHO, CO, NH_3_, SO_2_, CH_4_, H_2_S, NO_2_, NO	16	PCA, SVM	[299]
	ozone monitoring	O_3_, NO_2_, C_3_H_6_O, CH_2_O, C_2_H_6_O	7	PCA	[300]
	Detection of environmental pollution gases such as methane	CH_4_, CO, SO_2_, NH_3_	9	PCA	[301]
	air pollutant	CO, NO_2_, H_2_S, SO_2_	6	ANN	[210]
Indoor harmful gas	Indoor air pollution detection	HCHO, NH_3_	3	BPNN	[302]
	Indoor harmful gas anti-interference detection	CH_4_, C_2_H_5_OH, C_7_H_8_, VOCs, CH_3_CH_2_CH_3_, H_2_, CO, CH_2_O, C_4_H_10_, sulfide	6	LDA, PCA, BP-ANN	[181]
	The sources affecting indoor air quality were classified	CO_2_, VOCs, O_3_, NO_2_	4	ANN	[303]
	Odor monitoring in composting hall	C_2_H_5_OH, C_6_H_6_, CH_3_COCH_3_, H_2_, natural gas, CH_4_, CH_3_CH_2_CH_3_, C_4_H_10_, organic solvents, water	6	PCA, DFA, PLS	[304]
	Indoor pollution gas monitoring	C_6_H_6_, C_8_H_10_, C_7_H_8_, C_2_H_5_OH, CH_2_O, NO_2_	5	PCA	[171]
	Monitoring of indoor air pollutants	CH_2_O, C_6_H_6_, C_7_H_8_, CO, NH_3_, NO_2_	4	SFAM, MLP, FLDA, HSVM, SVM	[172]

#### Flammable and Explosive Toxic Gas

The concentration measurement of harmful gases is crucial as different gases have varying exposure thresholds. Continuous monitoring of these gases poses a significant challenge to enterprise safety, particularly in industries such as power, coal, and petrochemicals [167]. Hence, there is immense value in the timely and accurate detection and monitoring of these hazardous gases [168]. Multisensor arrays are commonly employed to detect concentrations of toxic gases in industrial settings, as well as flammable gases, and trace vapors released by explosives, or to assess the condition of explosives during storage.

**Fig. 10:**
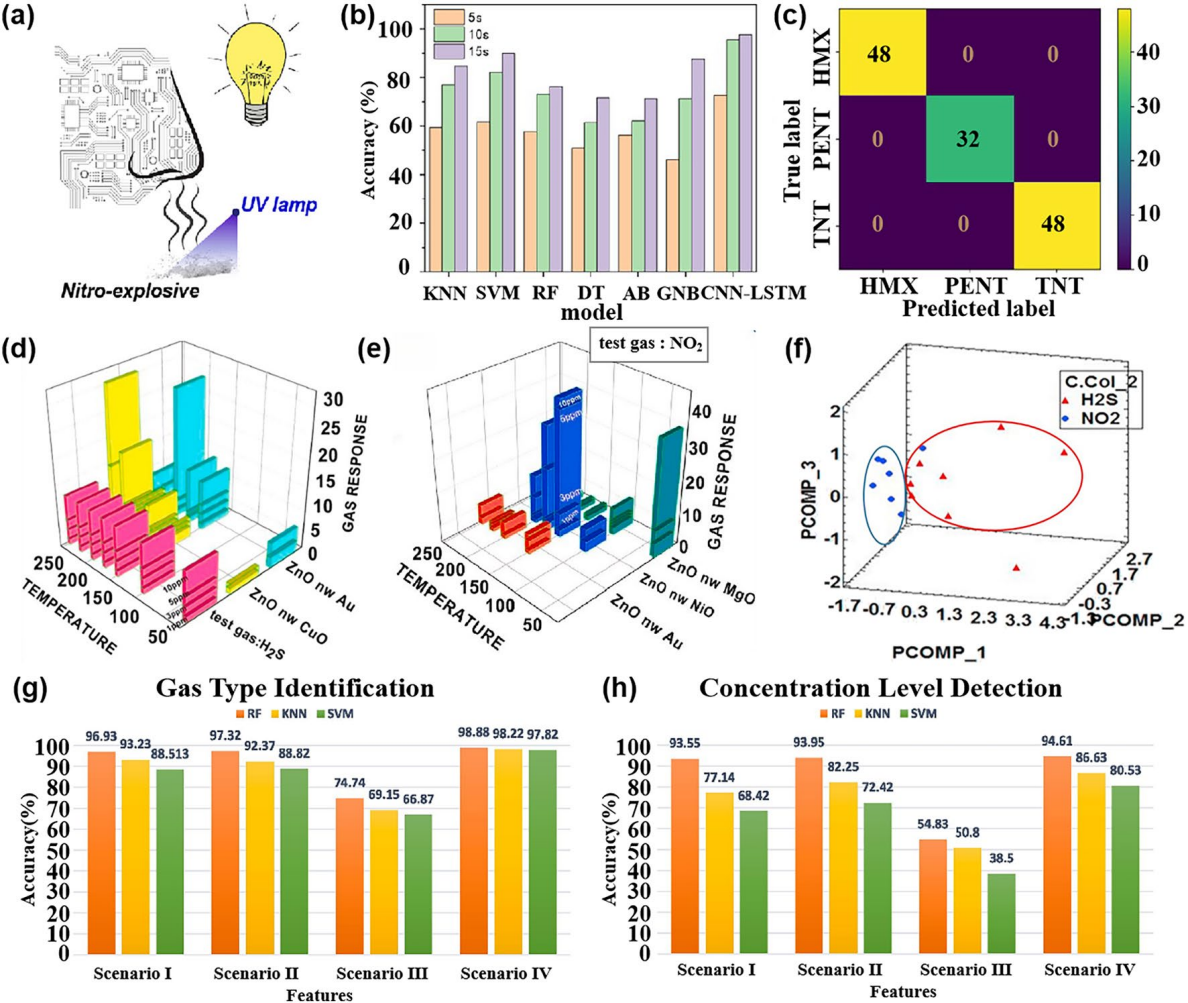
Pattern recognition methods in flammable and explosive harmful gas monitoring. **a** Sensitivity of the sensor array is enhanced by UV irradiation. **b** Accuracy of different models between 5, 10 and 15 s **c** Confusion matrix obtained by verifying all samples. Reproduced with the permission from Ref. [173], Copyright American Chemical Society 2023. **d**‒**e** Detection of toxic gases by E-nose based on ZnO nanowires Graphical analysis: Bar chart of MS towards H_2_S and NO_2_. **f** PCA was used to distinguish H_2_S and NO_2_. Reproduced with the permission from Ref. [177], Copyright Elsevier 2021. **g** Accuracy of the three algorithms corresponding to the gas type identification in four scenarios. **h** Accuracy of the three algorithms corresponding to the gas concentration level identification in four scenarios. Reproduced with the permission from Ref. [178], Copyright Elsevier 2022

Sensitive and selective detection of nitro explosive vapors, such as 2,4,6-trinitrotoluene (TNT), dinitrotoluene (DNT), and hexogen (RDX), remains a challenge due to their low concentrations (in parts per billion) and the extremely low vapor pressure of RDX (in parts per trillion) at room temperature [169]. In a recent study, Li et al. [170] successfully identified 11 types of military and improvised explosive vapors employing a TiO_2_ gas sensor array. These included 5 types of nitro-explosive vapors (TNT, DNT, para-nitro toluene (PNT), RDX, and picric acid (PA)) and 6 types of improvised explosive vapors (such as potassium nitrate, potassium chloride, potassium permanganate, sulfur, ammonium nitrate and urea). The identification of these vapors was achieved within 75 s at room temperature using PCA and fingerprint pattern recognition methods, which proved to be more efficient than capillary electrophoresis and ion mobility spectrometry [171, 172]. Furthermore, Liu et al. [173] proposed a novel method for rapid explosive trace detection by utilizing gas sensors exposed to ultraviolet light, as shown in Fig. 10a. This method successfully detected trace amounts of TNT, cyclotetramethylene tetranitroamine (HMX), and pentaerythritol tetranitrate (PETN) in the gas phase. Additionally, the accuracy of six common classification models was combined for sampling times of 5, 10, and 15 s (Fig. 10b). The confusion matrix of 128 samples was tested and the results demonstrate that all samples were accurately predicted (Fig. 10c). The accuracy of a one-dimensional CNN for rapid trace recognition of TNT, HMX, and PETN reached 97.7%, with a response time of within 15 s. However, the identification of explosive vapor based on MOS sensors needs to overcome problems such as nonlinear changes in concentration and drift caused by control circuits. For this reason, López et al. [174] proposed to use the transition region of the MOS signal as characteristic information to provide chemical vapor properties. Finally, combined with PCA and Mahalanobis distance for feature analysis, triacetone trioxide can be identified in real-time within the full steam concentration range of solid explosive spontaneous combustion emissions.

Similarly, the effective detection of flammable and hazardous gases during the industrial production process is crucial for ensuring the safety of workers and factories. Utilizing pattern recognition methods can aid in understanding environmental changes and enhance our ability to cope with them [175]. Attallah [176] proposes an intelligent detection device for industrial gas leaks, which consists of seven MOS sensor arrays and utilizes infrared thermal imaging. They also developed a multi-modal data fusion method based on three CNN models and bidirectional long-short memory to improve gas detection performance. The accuracy of intermediate and multitask fusion reaches 98.47% and 99.25% respectively, making it suitable for industrial harmful gas detection applications.

Sinju et al. [177] developed a novel ZnO NWs-based E-nose to qualitatively and quantitatively identify toxic gases NO_2_ and H_2_S by combining pattern recognition methods. The surface of hydrothermally grown ZnO NWs was modified with Au, CuO, NiO, and MgO to achieve multiple sensors. The experiments demonstrated that the sensor exhibited a significant response to H_2_S and NO_2_. Figure 10d, e shows the graphical analysis of the response of multiple sensors (MS) towards two distinct gases namely H_2_S and NO_2_. Using this graph, the data can be easily analyzed qualitatively. Furthermore, multivariate data analysis combined with PCA analysis was utilized to establish the correlation between measurement parameters (gas concentration, sensor response, response and recovery time of the developed MS). This correlation, as depicted in Fig. 10f, facilitated a clear separation between the target gases (H_2_S and NO_2_). Attallah and Morsi [178] proposed a smart E-nose system based on AI and feature selection to identify a variety of flammable and harmful gases and their concentrations. The features were extracted in the time domain, frequency domain, and time–frequency domain of the five MOS sensors, and their effects on the recognition performance were analyzed. As shown in Fig. 10g, the combination of time features with frequency and time–frequency features improved the performance of the proposed E-nose. For the identification of gas concentration, Fig. 10h demonstrates the accuracy of concentration prediction using different schemes, highlighting the superiority of combining multiple domain features over a single feature.

Improper use of combustible gases such as CO and CH_4_ can have severe adverse effects on human life, including the risk of fires and explosions. A study by Areej Shahid et al. [179] developed a sensor array based on SnO_2_ for detecting CO and CH_4_. The artificial neural network achieved a high classification accuracy of 98.7% for these toxic gases. Furthermore, the least squares regression (LSR) estimator achieved a minimum accuracy of 95.5% and 94.4% for CH_4_ concentrations of 50–1000 parts per million (ppm) and CO concentrations of 10–200 ppm, respectively. In 2020, Kang et al. [180] utilized five different metal oxides to create a high-throughput multi-sensor array and successfully classified seven target analytes (C_7_H_8_, NO, NH_3_, C_2_H_5_OH, C_3_H_6_O, C_6_H_14_) using PCA pattern recognition analysis. Moreover, Zhang et al. [181] proposed a semi-quantitative and synchronous anti-interference E-nose system based on a MOS sensor array to detect mixtures of 10–1000 ppm CO and 500–10,000 ppm CH_4_, with the addition of interfering gases H_2_ and CH_2_O to simulate more complex environments. They employed LDA, PCA, and BPNN for pattern recognition. The LDA model was still able to distinguish most levels even after adding interference samples, while the PCA method showed a high misjudgment rate, particularly for CO. Ni et al. [182] used a sensor array composed of 14 MOX gas sensors to predict the CO concentration under different conditions and proposed a new deep learning model Gaussian-TCN based on time CNN and Gaussian error linear unit. The original activation function is replaced by the Gaussian error linear unit of TCN to obtain better nonlinear performance. When Gaussian-TCN is used for regression prediction of CO concentration, the final result is better than TCN, LSTM, and GRU. It can be seen that Gaussian-TCN is a good technique for E-nose prediction analysis. Recently, Mao et al. [183] proposed a hybrid deep neural network (H-CRNN) combining CNN and RNN to predict CO concentration. A gated attention mechanism is proposed to enhance the reliability of H-CRNN to enhance the ability to capture key features. Compared with TCN, temporal pattern attention LSTM, space–time correspondence network, long- and short-term time-series network, LSTM and other algorithms, the average accuracy of the algorithm is as high as 96.42%, and the relative square error and relative absolute error are significantly reduced by 50.51%.

#### Indoor Harmful Gas

The improvement of life quality has led to an increasing demand for good indoor air quality, so the detection of indoor air quality has received significant attention, particularly concerning the presence of harmful gases such as benzene, toluene, ethylbenzene, and xylene. These gases can be emitted from sources such as smoking, building materials, furniture, and paint, making them difficult to detect. Studies have shown that prolonged exposure to such gases can increase the risk of cancer [184, 185]. Therefore, it is crucial to accurately detect and measure the concentrations of these specific harmful gases in indoor environments to safeguard human health.

**Fig. 11:**
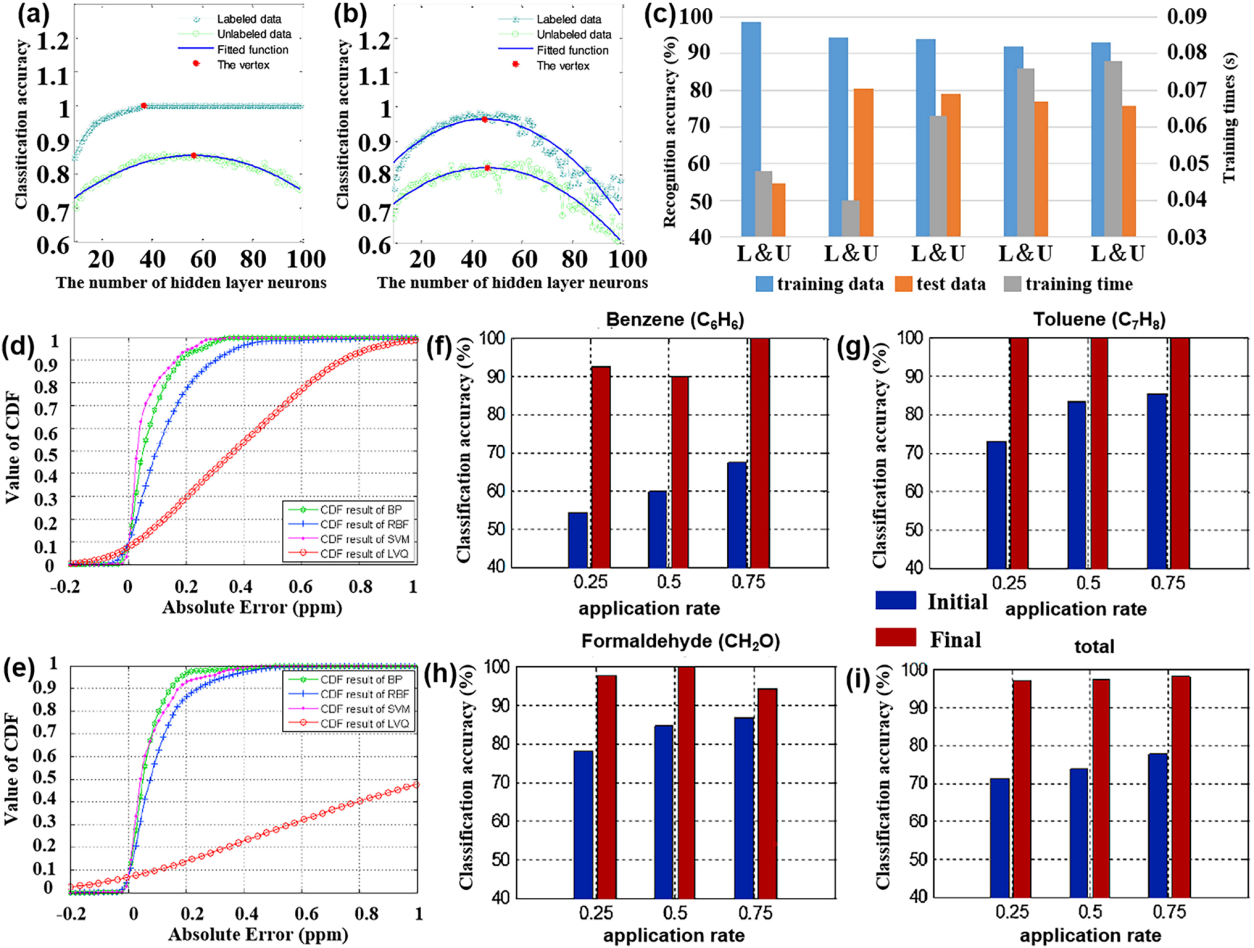
Application of pattern recognition method in indoor harmful gas detection. **a** Existing approach. **b** Presented approach. Reproduced with the permission from Ref. [186], Copyright Elsevier 2021. **c** Ratio of labeled and unlabeled samples from left to right was (1:9, 2:8, 3:7, 4:6 and 5:5) corresponding to the classification performance of LapSVM. Reproduced with the permission from Ref. [187]. Copyright Elsevier 2019. **d** Concentration of the test set is less than 1 ppm. **e** The concentration of the test set is greater than 1 ppm. Reproduced with the permission from Ref. [191], Copyright Elsevier 2017. **f‒i** Classification accuracy of the test data set for single different gases and mixed gases of C_6_H_6_, C_7_H_8_, HCHO and mixture of C_6_H_6_, C_7_H_8_ and HCHO. Reproduced with the permission from Ref. [156], Copyright Elsevier 2017

Zhang et al. [172] studied the potential applicability of E-nose combined with different data processing methods in the classification of six indoor air pollutants (HCHO, C_6_H_6_, C_7_H_8_, CO, NH_3_, and NO_2_). Compared with Euclidean Distance to centroid, simplified fuzzy adaptive resonance theory mapped network (SFAM), and MLP based on backpropagation learning rules, single fisher LDA, and single SVM, their proposed hierarchical SVM (HSVM) model has the best classification performance in three different training and testing ratios. Ma et al. [186] proposed a novel hidden neuron number optimization method, which uses the hierarchical clustering (HC) tree structure to reduce the complexity of the classifier training process. At the same time, the ANN based on ELM is used for classification at the same level. The improved hierarchical classifier was used to quantitatively test six toxic gases and three binary gas mixtures. In order to verify the performance of the proposed algorithm in selecting the number of optimal layer neurons, a single classifier ELM for sparse coding (ELM-SC) based on the ELM is used for verification. Figure 11a shows that the number of hidden layer neurons corresponding to the peak of the labeled sample and the unlabeled sample is different. Figure 11b shows that the classification accuracy of ELM-SC changes with the number of hidden layer neurons after applying the proposed algorithm. It can be seen that when the number reaches 45, the classification accuracy of labeled samples and unlabeled samples reaches the peak. The experimental results show that the classification accuracy of the hierarchical classifier is improved from 80% to 92% compared with the non-hierarchical classifier, which has better performance. Jia et al. [187] proposed a multi-core Laplacian SVM (LapSVM) algorithm to study the optimal ratio of labeled data to unlabeled data in E-nose training data, to achieve higher recognition accuracy. During the experiment, LapSVM, Twin SVM, RBFNN, and ELM were used to classify and identify four common indoor pollutants (C_6_H_6_, C_7_H_8_, HCHO, and CO). The classification performance of LapSVM under different proportions of labeled and unlabeled samples is described in Fig. 11c. When the training data is divided into 20% labeled data and 80% unlabeled data, the highest accuracy is obtained. The multi-core LapSVM achieves the best accuracy and the training time is shorter. At the same time, it is found that the higher the number of cores, the higher the accuracy.

In order to improve the performance of distinguishing four common indoor pollution gases(C_6_H_6_, C_7_H_8_, CH_2_O, and CO) based on an MOS multi-sensor array, Wang et al. [188] proposed an effective enhanced krill herd algorithm that is based on a novel decision weighting factor computing method. By optimizing the two parameters of SVM, they obtained a higher gas recognition rate. The experimental results demonstrate that the algorithm outperforms krill herd (KH), chaotic KH, quantum-behaved particle swarm optimization, particle swarm optimization (PSO), and GA. It proves to be an ideal optimization method for distinguishing indoor pollutant gases using a gas sensor array. Similarly, Fan et al. [189] introduced a clustering-based method (KmP algorithm) to address the lack of indoor harmful gases and interference source information in uncontrolled environments. During the experiment, they tested four data sets and compared the performance of the KmP algorithm with four classical clustering algorithms (Rodriguez–Laio, K-means, gaussian mixture model, and HC, with the KmP algorithm exhibiting stable and good gas recognition performance.

In order to detect low concentrations of indoor harmful gases, Leidinger et al. [190] developed an E-nose that operates under dynamic temperature conditions and utilizes LDA pattern recognition technology. This E-nose can detect dangerous VOCs levels as low as part per billion (ppb) and sub-ppb, enabling the detection of formaldehyde, benzene, and naphthalene. These three harmful gases are monitored to ensure indoor air quality. Similarly, He et al. [191] introduced a new type of E-nose for indoor air quality monitoring, which utilizes BPNN to process measurement data and predict formaldehyde concentration. Figure 11d, e shows the cumulative distribution function values of the absolute error of the concentration prediction of the four machine learning algorithms when the concentration of the test set is less than 1 ppm and more than 1 ppm, respectively. It can be seen that in either case, the performance of BP is usually better than that of SVM and radial basis function (RBF), while learning vector quantization (LVQ) performs poorly. In addition, they have designed an indoor air quality application service system to process, store, and display sensor data for users to help users master indoor air quality anytime and anywhere. Jiang et al. [156] proposed a RBFNN active learning algorithm based on improved committee query (QBC) (EQBC-RBFNN) and used it to distinguish three indoor pollutant gases (toluene, formaldehyde, and benzene). This technique effectively combines QBC and RBFNN with labeled and valuable unlabeled samples for E-nose training. Figure 11f–i describes the classification accuracy of benzene, toluene, formaldehyde, and three mixtures in the test data set. It can be seen that the recognition rate of these three gases, whether it is a single case or all three gases, has been improved to varying degrees.

#### Outdoor Harmful Gas

With the acceleration of urbanization and industrial development [192], as well as the increasing demand for transportation energy consumption [193], the emissions of atmospheric pollutants, including NO_x_, SO_x_, CO_x_, O_3_, and particulate matter, are also increasing. Even when the concentration of these harmful gases is only slightly higher than one million, it can have a serious impact on human health [194]. Therefore, effective monitoring of atmospheric pollutant emissions can greatly contribute to environmental protection and human health [195, 196]. Improving the sensitivity and selectivity of gas sensors is crucial for applications like air quality monitoring.

**Fig. 12:**
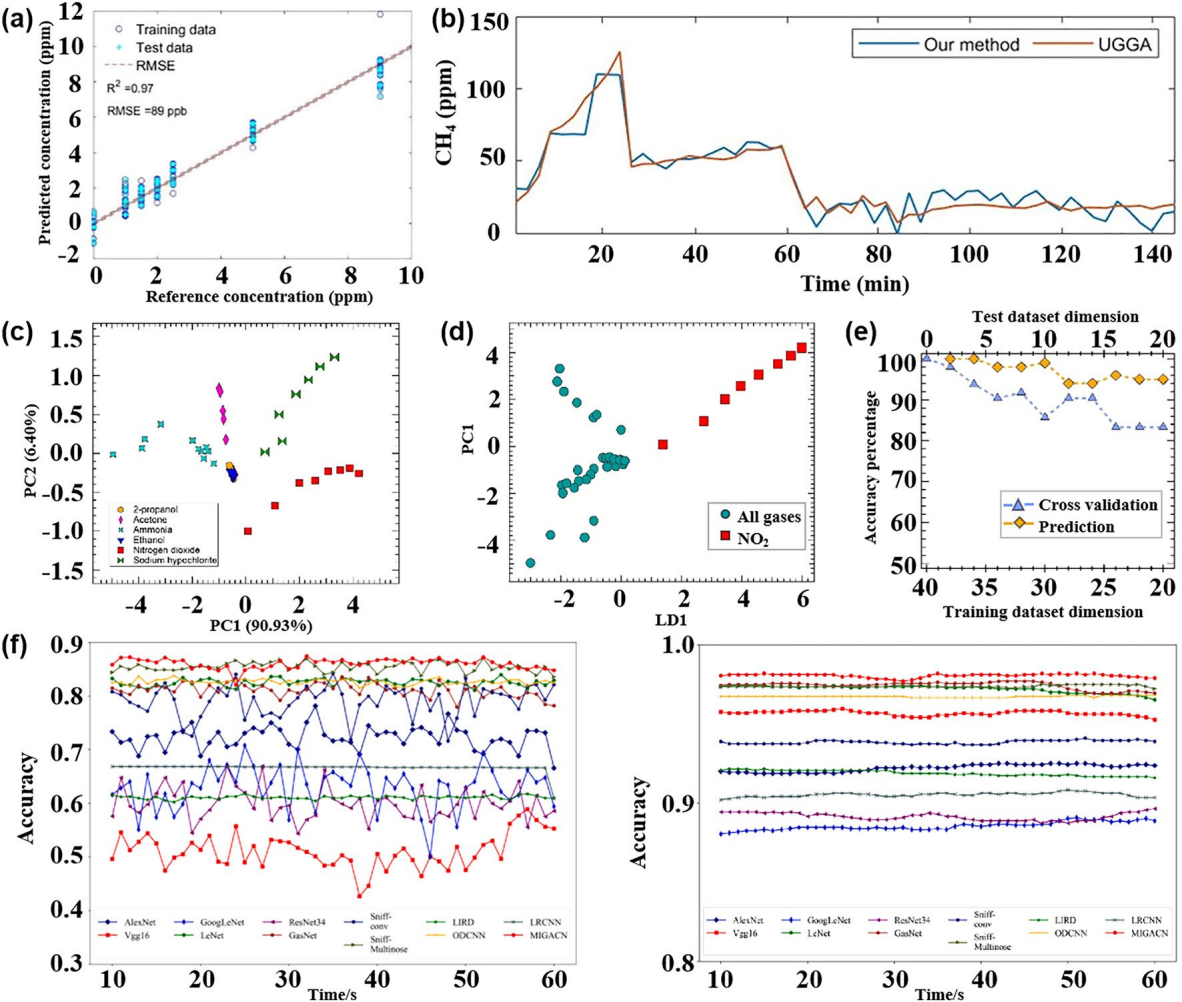
Application of pattern recognition methods in air pollutant detection. **a** PLSR was used to predict methane concentration under different concentrations of water vapor. **b** Change of methane with time in the model. Reproduced with the permission from Ref. [197], Copyright American Chemical Society 2024. **c** PCA results of six gases (NO_2_, NH_3_, NaClO, C_3_H_6_O, C_2_H_5_OH and C_22_H_47_NO). **d** Results of LDA on NO_2_ and all gases over the entire data set are plotted. **e** Cross-validation and prediction of the accuracy of training test sets for different dimensions. Reproduced with the permission from Ref. [157], Copyright Wiley 2023. **f** Average dynamic detection accuracy of each model before and after data enhancement is added. Reproduced with the permission from Ref. [201], Copyright Elsevier 2024

Reducing greenhouse gas methane is important for mitigating climate change. Domènech-Gil et al. [197] proposed the use of multiple types of sensors to obtain data, and the PLSR model prepared by tenfold cross-validation was used for quantification. Figure 12a shows the PLSR results of methane at different water vapor concentrations. Figure 12b shows that the method produces effective time prediction. Their work provides a fast, effective, and low-cost method to detect changes in methane concentration and verify the effectiveness of mitigation measures.

NO_2_ not only poses a threat to human health but also contributes to environmental hazards such as smoke and acid rain [198]. Another E-nose developed by Sayago et al. [199], was designed to detect NO_2_ at concentrations below 0.5 ppm, particularly between 150 and 200 °C. Impressively, it exhibited high responsiveness even at room temperature, making it suitable for monitoring low concentration gases. This development is expected to provide valuable insights into subtle changes in air pollution. Building upon the concept of artificial intelligence, Arroyo et al. [158] introduced an E-nose integrated with digital gas sensors for monitoring various concentrations of nitrogen oxides. They employed a multi-sensor neural network to classify sensor data in smartphone applications. Freddi et al. [157] utilized an E-nose to track and distinguish NO_2_ for environmental safety monitoring. Through PCA and LDA analysis, they were able to effectively differentiate between gases, achieving a prediction accuracy of over 95% for NO_2_ even in the presence of gas mixtures. As shown in Fig. 12c, the PCA results showed that NO_2_, NH_3_, NaClO, and C_3_H_6_O could be well classified, with only significant overlap betweenC_2_H_5_OH and C_22_H_47_NO. Figure 12d shows the results of two categories using LDA, and the cross-validation accuracy of the data set is 100%. In addition, they also studied the influence of the dimension of the training live test data set on the classification accuracy (Fig. 12e), where the prediction accuracy was 95%–100%, and the accuracy of cross-validation was 83%–100%. They also explored the possibility of enhancing the gas sensing performance of the array by improving the data set, the test set, and employing other analytical methods like neural networks.

In addition to nitrogen oxides, SO_2_ is also a common atmospheric pollutant. It is typically released into the atmosphere during processes such as fossil fuel incineration and sulfide ore metallurgical processing [198]. Inhalation of SO_2_, whether long-term or short-term, can have serious impacts on human health, particularly causing respiratory, lung, and eye injuries [200]. Developing gas sensors with high sensitivity, strong selectivity, and corrosion resistance remains a significant challenge. However, selecting appropriate pattern recognition methods can contribute to the analysis of specific gases.

Zhai et al. [201] proposed a novel convolutional network (MIGACN) model based on multi-level staggered group attention and responded to 10 industrial pollution gases including C_2_H_4_O, SO_2_, CHS, H_2_S, HCl, CO, NH_3_, NO, H_2_, and NO_2_ through a self-made E-nose system composed of 15 MOS gas sensors. Two feature learning modules are proposed to extract features at the time level and the sensor level, respectively. In addition, the data enhancement module is introduced to avoid the problem of insufficient training due to the small amount of gas sensor data. Figure 12f shows the classification accuracy of different algorithms before and after adding the data augmentation module. It can be seen that MIGACN has better classification performance and can achieve the highest average dynamic detection accuracy of 98.19% after adding the data augmentation module. The gas dynamic detection and identification problem in the future mixed gas scene provides a new solution. Environmental monitoring, particularly real-time gas detection, is crucial for maintaining air quality and ensuring public safety. Gas sensors play a significant role in this domain, but they face several challenges, especially in real-time applications. The response time of gas sensors is critical for real-time monitoring. Traditional gas sensors often have slower response times, which can impede their effectiveness in dynamic environments. This issue is particularly relevant in applications requiring immediate detection and action.

Burgués's team integrated their previously designed portable E-nose onto a drone, using dynamic sensor signals under flight conditions to train machine learning models for real-time odor measurement at a wastewater treatment plant [202]. This E-nose is equipped with 21 MOS sensors, a global positioning system receiver, and a radio communication system for real-time data transmission [203]. Measurement signals are sent to the base station every 6 s, where a designed PLS model combined with variable importance in projection scores analyzes the data in real time. This approach offers higher accuracy compared to traditional steady-state calibration methods. Recent advancements in deep learning have significantly improved the real-time capabilities of gas sensors, particularly E-nose systems. Deep learning models, such as CNN and RNN, enable real-time processing and prediction by allowing streaming data input and automatic feature extraction. For instance, Kang et al. [204] employed a 10-s time window, compressing gas sensor data collected over 10 s into 1 s for input into a CNN, enabling real-time data analysis. They achieved real-time detection and analysis of six gases, with a minimum response time of 1 s for CO and an accuracy of 98%. Similarly, Lee et al. [205] used a CNN model to detect ethanol and NO_2_ within 10 s and acetone and methanol within 30 s. The multi-task convolutional neural network designed by Wang et al. [206] only needs to input the response data for several seconds into the model to distinguish the prediction of gas type, concentration, and state. The model uses data from more than 10 million sensors for training. When the baseline is automatically tracked, 12 VOCs can be predicted by inputting 4 s data during the electronic nose response period, with an accuracy rate of up to 95%. The system can achieve real-time output of the results.

With the rapid advancements in device integration, IoT, and data processing technologies, combining E-nose technology with IoT system design promises to significantly enhance the efficiency of detecting hazardous gases. For instance, Abdullah’s team [207] developed a cloud-based laboratory air quality monitoring system, deploying E-noses as nodes across five different laboratories to collect real-time air pollutant data. This data is transmitted to a network server, where the cloud-based system processes, analyzes, and displays the results in real time.

Another team [208] developed an edge computing IoT device that supports wireless field monitoring platform interfaces and introduced an environmental adaptive continuous learning (EACL) method. Experimental validation with hazardous gases demonstrated that EACL could reduce training cycles by a factor of three and improve efficiency by 25%. In edge computing, data processing can be performed directly on the microcontroller unit (MCU) embedded in the E-nose. These compact devices can independently perform sensing, data collection, and processing, eliminating the dependence on external facilities, reducing hardware costs, and enabling real-time measurements.

Wang et al. [209] developed a gas detection microsystem comprising an application-specific integrated circuit chip and MEMS gas sensors to detect various odor components, such as ethylene glycol and ammonia. This integrated microsystem boasts high sensitivity, flexibility, and portability. Wang’s team [110] also designed an E-nose system with eight MEMS sensors, consisting of a sensor array, front-end analog circuit, MCU subsystem, communication subsystem, and power management unit. The MCU can process data from mobile devices while performing edge computing. Additionally, the E-nose features multiple communication interfaces, including universal serial bus (USB) and Bluetooth. By integrating onboard edge computing with the ANN model, this system can quantitatively identify 15 types of VOCs. The fault tolerance of this group-based E-nose is enhanced by at least tenfold compared to array-based E-noses. In the same year, Kwon et al. [210] implemented pattern recognition algorithms within an edge computing environment to alleviate the overload on monitoring servers. These studies contribute significantly to the development of advanced next-generation gas sensors with improved communication and monitoring systems.

### Medical Diagnosis

From a clinical perspective, endogenous VOCs produced through internal metabolic processes can serve as valuable indicators for disease detection. Numerous studies have demonstrated that analyzing VOCs in blood, urine, feces, and exhaled gases can help differentiate between different diseases. VOCs are gaseous molecules that can be easily and non-invasively sampled from breath [211]. Currently, respiratory analysis is being utilized in medicine and clinical pathology as a non-invasive method to assess an individual's health status. The pattern recognition method, employing a multi-sensor array, is used to diagnose various diseases. These include liver diseases, diabetes, asthma, as well as cancers such as lung cancer, prostate cancer, rectal cancer, advanced adenoma, breast cancer, head and neck cancer, ovarian cancer, and bladder cancer. However, endogenous VOCs typically exist in concentrations ranging from 1 to 5000 ppb, and there is a wide variety of gases [212]. Detecting the specific marker VOCs to confirm a disease remains a significant challenge. Consequently, researchers are increasingly focused on identifying suitable pattern recognition methods for analyzing auxiliary sensor response data to develop fast, non-destructive, efficient, and reliable diagnostic instruments for diseases. This section will discuss the application of multi-sensor arrays combined with pattern recognition methods in disease diagnosis, specifically focusing on malignant tumors, typical respiratory diseases, and metabolic diseases. Table 5 encapsulates the sophisticated deployment of pattern recognition algorithms for addressing cross-sensitivity challenges in disease diagnosis, detailing the specific algorithms employed, their corresponding target gases, and the comprehensive count of gas sensor arrays implemented.

**Table 5:** Summarizes and describes the different pattern recognition methods used in disease diagnosis

Species	Purpose	Detectable gases	Sensor Number	Pattern recognition	References
Cancer	diagnosis of lung cancer	C_2_H_5_OH, H_2_, C_4_H_10_, C_7_H_8_, H_2_S, NH_3_, CO, CH_3_COCH_3_, C_8_H_10_, combustible gases	11	PCA-SVE	[305]
	diagnosis of lung cancer	Alkanes, CO, NH_3_, H_2_S, NO, CH_4_, VOCs, H_2_	6	PCA, KNN, LDA, SVM, RF, logistic regression	[217]
	Distinguishing lung cancer from COPD	CH_4_, C_4_H_10_, C_2_H_5_OH, CH_3_COCH_3_, C_6_H_6_, NH_3_, CO, CH_3_CH_2_CH_3_, H_2_	5	SVM	[306]
	Diagnosis of colorectal cancer	Aromatic compounds, organic compounds, H_2_, CH_4_, C_2_H_5_OH, aliphatic compounds	10	Random forest, neural network	[307]
	Distinguish between colorectal cancer and control group	CH_3_COCH_3_, C_2_H_5_OH, C_4_H_8_O_2_,4-methyl octane	–	DFA	[308]
	Diagnosis of head and neck cancer	CH_4_, CO, NO_x_	4	Logistic regression	[309]
	Diagnosis of ovarian cancer	Decanal, nonanal, styrene, 2-butanone, hexadecane	–	DFA	[310]
Respiratory diseases	COVID-19 detection	CO, C_2_H_5_OH, H_2_, H_2_S, C_4_H_10_, CH_4_, NH_3_, C_6_H_6_, C_6_H_14_, CH_3_COCH_3_, hydrofluorocarbons, C_7_H_8_, CH_3_CH_2_CH_3_	10	HAC, DT	[68]
	Fast COVID-19 screening	C_3_H_8_O, CH_3_CH_2_CH_3_,2-butanone cluster, 2-butanone, methanol monomer and dimer, CH_3_CHO, CH_3_COCH_3_, octanal, isoprene, C_7_H_16_	10	LDA, SVM, MLP, DNN	[235]
	COPD Detection	CH_4_, C_4_H_10_, C_2_H_5_OH, CO, CH_3_COCH_3_, C_6_H_6_, NH_3_, H_2_, C_4_H_10_, CH_3_CH_2_CH_3_	5	SVM, K-NN, Naive Bayes, LDA, logistic regression	[311]
Metabolic diseases	Identification of myocardial infarction and coronary artery disease	O_3_, H_2_S, CO, SO_2_, NO_2_, CH_4_, C_2_H_5_OH, C_4_H_10_, H_2_, CH_3_CH_2_CH_3_, NH_3_, C_7_H_8_, O_2_, solvent vapors, C_3_H_9_N, CH_3_SH, CFCs	19	SVM, K-NN, ANN	[312]
	Distinguishing patients with cirrhosis	C_2_H_5_OH, C_6_H_6_, NH_3_, NO, CH_3_CH_2_CH_3_, H_2_, CH_4_, C_7_H_8_, CO_2_, CH_2_O, CH_3_COCH_3_, CH_4_O	5	PCA, DFA, SVM	[248]
	Identification of diabetic patients and healthy individuals	NO, CH_2_O, C_2_H_5_OH, C_6_H_6_, CH_3_CH_2_CH_3_, H_2_, CH_4_O, CO_2_, CH_3_COCH_3_, C_7_H_8_, CH_4_, NH_3_	5	PCA, DFA, SVM	[313]
	Distinguish between malignant and non-malignant gastric diseases	Furfural, isoprene, 2-butoxy-ethanol, 6-methyl-5-hepten-2-one, 2-propenenitrile	12	DFA	[314]

#### Cancer

Cancer is a significant public health issue worldwide. In 2020, the American Cancer Society reported over 1.8 million cancer cases in the United States. The four major cancers are lung cancer, colorectal cancer, breast cancer, and prostate cancer [213]. Medical studies have established a connection between the presence and growth of tumors and the emission of VOCs resulting from cell membrane peroxidation or cell metabolism. These VOCs can be detected either directly from the top space of cancer cells or through exhaled respiration. Therefore, the analysis of human exhaled gas plays a crucial role in predicting the progression of cancer in patients who exhibit few early indicators and slow progression [214].

**Fig. 13:**
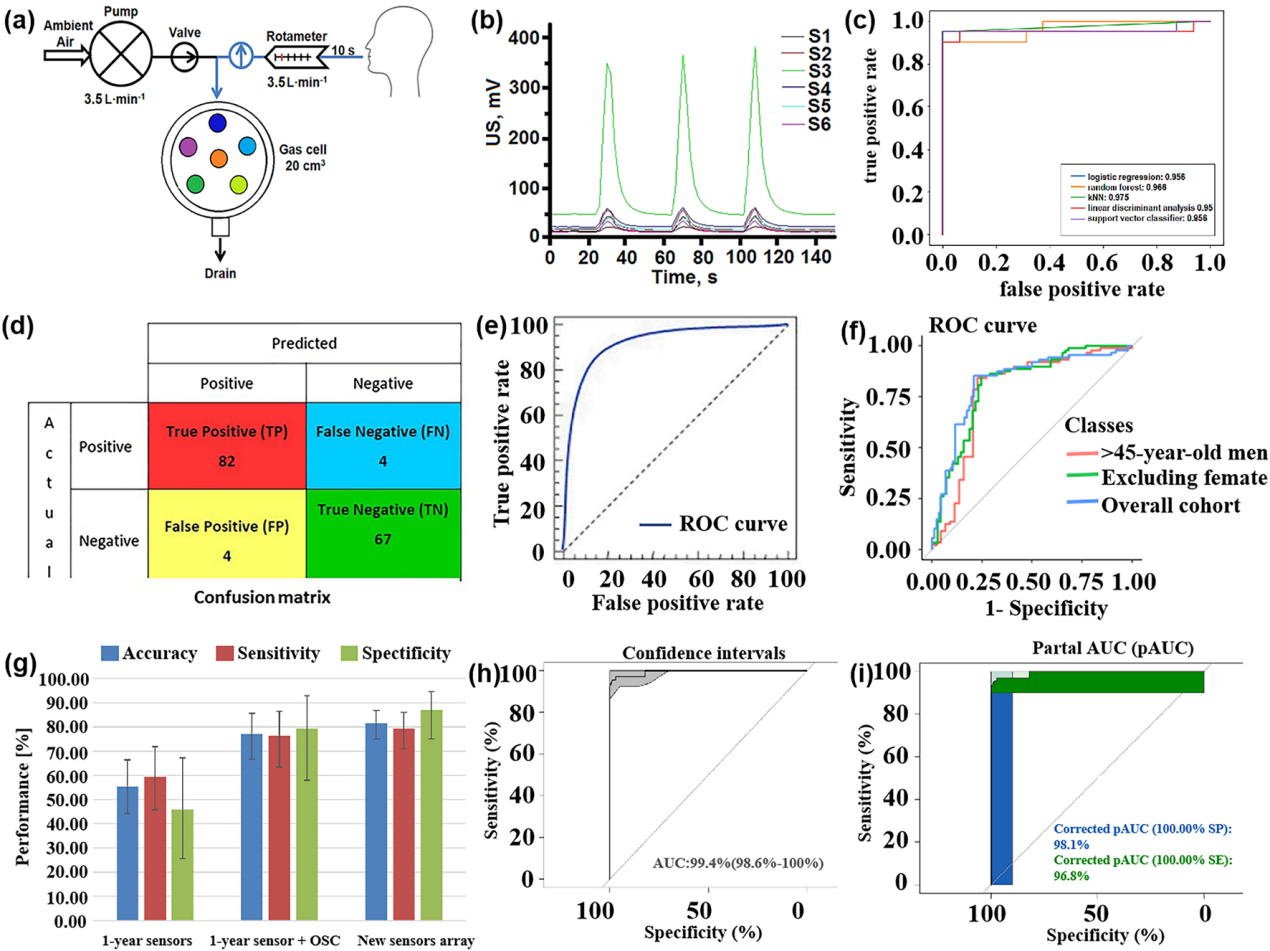
Application of pattern recognition methods in lung cancer diagnosis. **a** Working diagram of online respiratory analysis setting. **b** Continuous response of six sensors to exhaled gas samples (S1-S6 represents the six sensors used in the experiment). **c** Five models correspond to ROC curves. Reproduced with the permission from Ref. [217], Copyright IOP Publishing 2020. **d** Confusion matrix of 157 samples. **e** ROC curve of 157 samples. Reproduced with the permission from Ref. [220], Copyright Elsevier 2018. **f** Diagnostic accuracy report of E-nose. Reproduced with the permission from Ref. [227], Copyright Wiley 2022. **g** Comparison of classification performance obtained by internal cross-validation of new sensor arrays and old sensor arrays before and after drift correction (error line = 95% confidence interval). Reproduced with the permission from Ref. [228], Copyright Elsevier 2022. **h** Discriminatory accuracy is expressed as AUC with a 95% confidence interval. **i** The partial area under the receiver operating curve (pAUC). Reproduced with the permission from Ref. [232], Copyright Nature 2021

Lung cancer, the leading cause of cancer death worldwide, is a primary malignant tumor of the lung [215]. Researchers have explored the use of endogenous VOCs, including hydrocarbons, primary alcohols, secondary alcohols, aldehydes, and branched aldehydes, ketones, esters, nitriles, and aromatic compounds, as potential biomarkers for studying lung cancer. For instance, Shlomi et al. [216] conducted a study using a set of arrays with 40 sensors to analyze exhaled gas and diagnose epidermal growth factor receptor mutations in lung cancer patients. The results showed that exhaled gas analysis had a high accuracy (about 88%) in identifying patients with early-stage lung cancer and benign pulmonary nodules, supporting the use of VOCs as markers for lung cancer diagnosis. However, it should be noted that the concentration of VOCs in clinical respiratory samples is typically at the ppb level. To address this challenge, the performance of MOS-based sensor arrays can be enhanced by utilizing pattern recognition algorithms for optimized data analysis. For instance, Kononov et al. [217] employed an array of six metal oxide gas sensors to analyze exhaled gas samples from 118 volunteers, including 65 individuals in the lung cancer group and 53 in the healthy group. The respiratory analysis program is depicted in Fig. 13a. The response of the sensor, representing an online mode peak (Fig. 13b), was selected by the authors as the analysis signal. Figure 13c illustrates five different models used for analysis, displaying the receiver operating characteristic (ROC) and area under the curve for each model. The findings indicate that logistic regression and K-NN classification models exhibited superior performance in distinguishing lung cancer patients from healthy controls. De Vries et al. [218] tested the prospective discovery of early lung cancer in chronic obstructive pulmonary disease (COPD) patients using E-nose technology. By using PCA, all PCs with eigenvalues greater than 1 are retained, and the data dimension is reduced to prevent the risk of overfitting. Then, three machine learning methods including gradient hoist, adaptive minimum absolute shrinkage and selection operator, and sparse PLS discriminant analysis were used to classify the data after dimensionality reduction. The results showed that the accuracy of distinguishing COPD patients from lung cancer patients was 87%, the sensitivity was 86%, and the specificity was 89%. It provides a fast method for early identification of COPD patients with malignant tumors.

Colorectal cancer (CRC) is a common type of tumor. Previous research has demonstrated that chemical resistance gas sensors can detect tumor gas markers at concentrations as low as 10 ppb or even 0.1 ppb [219]. By utilizing gas sensors to detect colorectal cancer biomarkers, it is possible to screen and detect tumors early, preventing them from progressing into malignant cancers and improving the chances of a cure. Zonta et al. [220] used E-nose and fecal occult blood tests to distinguish CRC patients from healthy people. In order to find the best gas sensor combination, they tested 19 different materials including metal oxides and sulfides and tested them on 157 samples. Figure 13d, e are the confusion matrix and ROC curve after PCA analysis, respectively. The ROC curve shows 95% sensitivity and specificity. In addition, after selecting five sensors, PCA and SVM were combined to find that all CRC patients and 98% of healthy subjects in the test samples could be identified. Similarly, Zonta et al. [221] introduced a non-destructive testing device called SCENT A1, which is based on semiconductor gas sensors. They analyzed 398 samples of fecal odor from colorectal cancer patients and healthy individuals using machine learning techniques. The SVM-based algorithms yielded average sensitivity and specificity values of 84.1% and 82.4%, respectively.

Prostate cancer (PCa) is a prevalent malignant tumor in men worldwide, with its incidence increasing year by year [222]. According to the global cancer statistics of 2018, there were approximately 1,276,106 new cases of PCa and 358,989 deaths worldwide [223]. Prostate cancer is typically asymptomatic and slow-growing. However, if detected early, the cure rate is very high [224]. Traditionally, a prostate biopsy is required to confirm the presence of cancer. Unfortunately, prostate biopsy is an invasive examination that carries significant risks and increases patient management costs [225]. Therefore, there is a need for more reliable and non-invasive methods to diagnose prostate cancer. Numerous studies on biomarkers of prostate cancer have been published. Laura Capelli et al. [226] analyzed the performance of an E-nose in diagnosing prostate cancer patients by analyzing the volatiles extracted from the urine of 132 PCa patients and 60 control groups. They used Boruta algorithm and RF algorithm to construct a PCa classification model, achieving 83% accuracy, 82% sensitivity, and 87% specificity. It is believed that E-nose can be used for rapid and non-invasive diagnosis of PCa, showing high accuracy and promising prospects for the future, further clinical trials are needed to verify its application in PCa diagnosis. In a prospective study conducted by Taverna et al. [227], the accuracy of an E-nose in identifying prostate cancer in urine samples was evaluated among patients with continuous PCa. The E-nose utilized six n-type doped MOS sensors printed by inkjet printing, which differed from the active layer. TiO_2_, ZnO, and SnO_2_ were the sensors used for urine analysis. The E-nose recorded the typical response signals, and an RF pattern recognition model was established for analysis. The diagnostic accuracy of E-nose is reported as the area under the ROC 0.821 (95% CI 0.764–0.879) (Fig. 13f). Bax et al. [228] think that the problem of sensor drift may be one of the reasons why the E-nose has not yet been introduced into clinical practice. To this end, they proposed a data processing protocol to compensate for drift. It was applied to the urine headspace data set of 81 prostate cancer patients and 41 healthy people obtained within 9 months. The experimental results are shown in Fig. 13g. Comparing the classification performance of the three sensor arrays (old and new sensors and old sensors for drift compensation), it can be seen that the accuracy is restored from 55% to 80%, which effectively reduces the drift of the old sensor for 1 year. Durán Acevedo et al. [229] used E-nose and E-tongue equipment to analyze urine and exhaled gas samples to detect prostate cancer. The control group included benign prostatic hyperplasia, prostatitis group, and healthy group. Different machine learning methods are used, such as QDA, naive Bayes, SVM, K-NN, RF, and DT. The classification accuracy of E-tongue and E-nose was 92.9% and 100%, respectively.

Breast cancer is the most common cancer among women and the leading cause of cancer death. However, current image-based techniques like mammography cannot accurately identify tumors in women with fibrosis-cystic mastopathy, leading to a high number of false positives. Fortunately, the evaluation of diseases like breast cancer has been extensively studied using human VOCs. For instance, Lavra et al. [230] conducted an in vitro study demonstrating that VOCs analysis can provide clinically relevant information about proliferative and molecular features of breast cancer cells. They also developed a partial least squares-discriminant analysis (PLS-DA) model to classify the signals of temperature-modulated MOS gas sensors, which lays the foundation for the development of low-cost cancer diagnostic equipment. The pattern recognition method is crucial in the gas classification ability of the system. The experimental results obtained by Judith Giro Benet et al. [231] demonstrate that the introduction of a neural network has a significant impact on the classification of E-nose data. This leads to an increase in the model’s accuracy from 58.3% to 75%. Although the overall accuracy is not optimal, the author suggests that better classification results could be achieved with a larger sample size, particularly with the number of training samples for the CNN model. In a separate study, Yang et al. [232] collected alveolar air samples from both breast cancer patients and non-cancer patients. They utilized RF to develop prediction models for breast cancer and molecular phenotypes. The 95% confidence interval of the ROC for 2000 replicates using bootstrap resampling is shown in Fig. 13h. Figure 13i shows the partial area under the working curve (pAUC) for 899 subjects, the prediction accuracy was 91%, the sensitivity was 86%, and the specificity was 97%. Among them, the positive predictive value was 97%, and the negative predictive value was 97%. This study demonstrates high accuracy and reliability in the identification of breast cancer and molecular subtypes, indicating great potential for the future development of rapid breast cancer diagnostic tools.

#### Respiratory Diseases

Respiratory diseases encompass conditions that affect the airways and lungs involved in human breathing. Common examples include asthma, pneumonia, and respiratory infections like the coronavirus disease that emerged in 2019. The widespread occurrence of certain epidemic respiratory diseases can have significant impacts on both the economic development of society and the overall health of the population. Consequently, there is a growing demand for large-scale real-time detection tools. Analyzing exhaled gas patterns offers a promising approach to disease detection and classification. For instance, Nakhleh et al. [233] employed artificial intelligence nanoarrays to analyze respiratory samples from 1404 individuals, diagnosing and classifying 17 diseases. To explore the correlations or distinctions among different diseases, HCA was utilized, revealing substantial similarities between subgroups sharing common pathophysiology. Subsequently, the gas information obtained from the sensor array was qualitatively or quantitatively analyzed using pattern recognition methods, enhancing its practical application in the diagnosis of human diseases. Kwiatkowski et al. [234] conducted a study using gas sensors to detect exhaled gas from 33 COVID-19 patients and 17 healthy volunteers in local hospitals. The study utilized four algorithms, namely neural network, RF, KNN, and SVM, for analysis. The classification accuracy of all algorithms exceeded 91%, with the ability to detect COVID-19 patients reaching 80% even in the worst-case scenario.

**Fig. 14:**
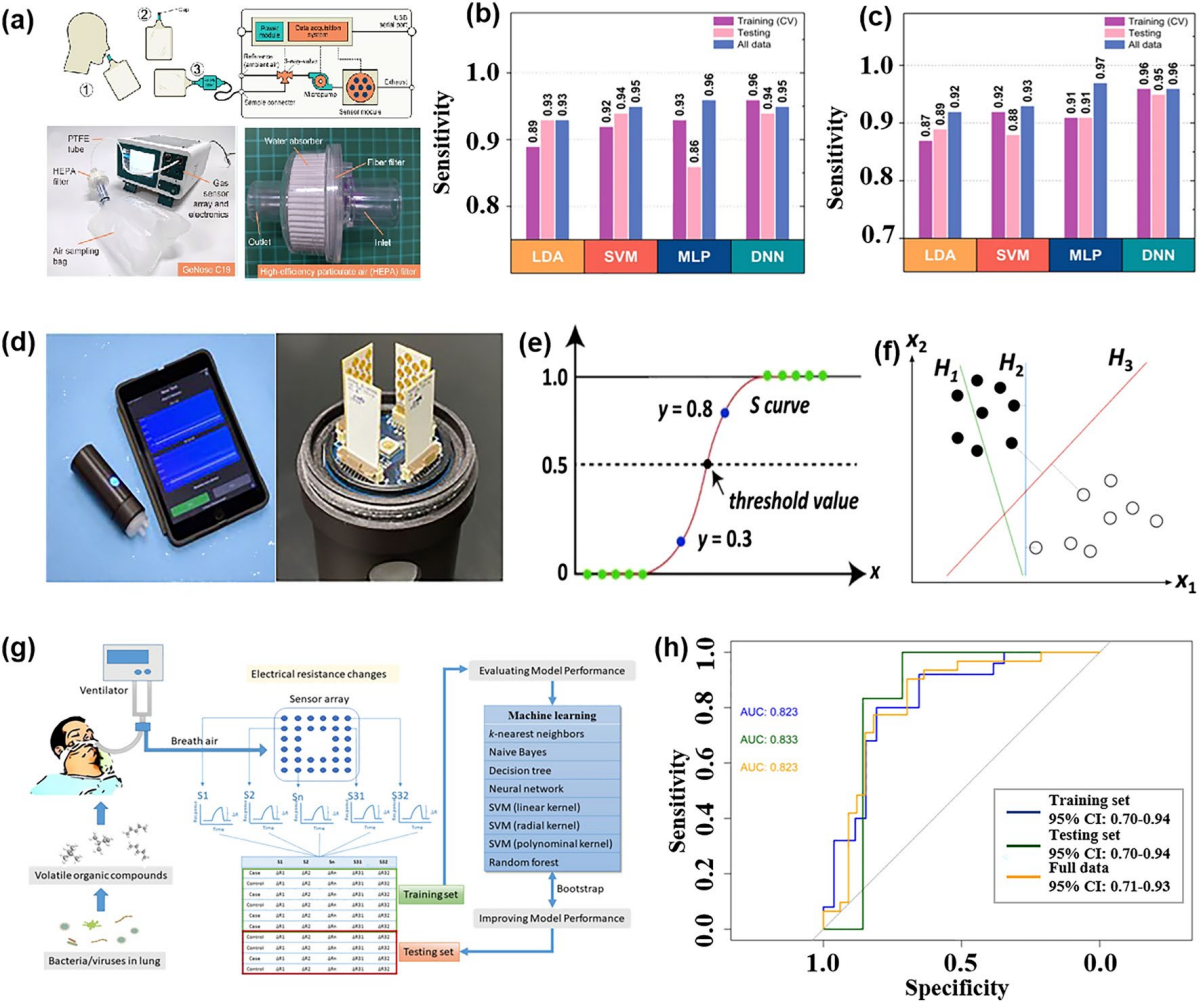
Application of pattern recognition method combined with a multi-sensor array in the diagnosis of respiratory diseases. **a** Integrated E-nose system and its components for detecting COVID-19. **b, c** Four machine learning algorithms (LDA, SVM, MLP, and DNN) are used to analyze the sensitivity and specificity histograms obtained from the training and the test sample data sets, respectively. Reproduced with the permission from Ref. [235], Copyright Springer Nature.2022. **d** Integrated E-nose system includes four 16-sensor arrays. **e** Logistic regression model. **f** SVM model. Reproduced with the permission from Ref. [237], American Chemical Society 2023. **g** Establish a standardized protocol based on machine learning algorithms and its implementation process. **h** AUC curves of the training set, test set, and all data sets. Reproduced with the permission from Ref. [238], Copyright Springer Nature.2020

Another study by Nurputra et al. [235] employed four different machine learning methods (LDA, SVM, stacked MLP, and deep neural network (DNN)) to test 615 respiratory samples for evaluating the performance of an E-nose in rapidly identifying COVID-19. The study also included respiratory samples from 40 COVID-19 patients and healthy controls. The integrated E-nose system for detecting COVID-19 is illustrated in Fig. 14a. Furthermore, Fig. 14b, c presents the sensitivity and specificity of the four machine learning algorithms for both the training set and the test set. Notably, the DNN model demonstrated the best results, achieving a sensitivity of 95.5% and specificity of 95.7%. These findings provide support for the utilization of E-nose technology in large-scale COVID-19 screening and monitoring for medical efficiency. In a separate study, Bhandari et al. [236] designed a mechanical lung ventilation device using a sensor array for monitoring severe COVID-19 patients in a mechanical lung ventilator. This array includes an environmental sensor measuring temperature, pressure, and relative humidity, as well as a series of chemical resistance MOX gas sensors that detect VOCs (methylpent-2-enal, 2,4-octadiene, 1-chloroheptane, and nonanal) patterns in the patient's breath. The sensor device is installed on the ventilation exhaust port for real-time monitoring and displays stable readings. The PCA separation is satisfactory. However, further research is needed to enhance the sensor data set in order to accurately infer the severity of the patient's condition. Li et al. [237] designed a cylindrical E-nose device that integrates a resistivity-based nanosensor, a wireless USB interface, and a rechargeable battery, and the device can also be connected to a tablet or mobile phone to display data in real time (Fig. 14d). Through this new electronic nose device, human exhaled VOC was analyzed, and logistic regression (Fig. 14e) and SVM model (Fig. 14f) were established for classification, so as to realize on-site rapid screening of COVID-19 infection. In Fig. 14e, green dots represent true label values, while blue dots denote predicted values by the model. In Fig. 14f, black and white dots represent two different label values.

Rianne de Vries et al. [218] conducted a study using SpiroNose to analyze exhaled breath and evaluate its diagnostic accuracy in differentiating between COPD patients and lung cancer patients. Additionally, the study aimed to predict early lung cancer in COPD patients. The signal processing of the E-nose involved various steps such as detrending, filtering, ambient air correction, automatic peak detection, and parameter selection. Four machine learning methods were employed to classify the E-nose data. The findings revealed a cross-validation accuracy of 88% and an AUC of 0.93. This study demonstrates that pattern recognition methods in exhaled breath analysis can effectively distinguish between lung cancer and COPD.

Chen et al. [238] utilized machine learning techniques to analyze respiratory gases for the detection of ventilator-associated pneumonia (VAP), as depicted in Fig. 14g. They employed eight machine learning algorithms, including K-NN, naive bayes, DT, neural network, SVM, linear kernel, polynomial kernel, radial basis kernel, and RF, to construct prediction models. The AUC values for the training set, test set, and all data sets are presented in Fig. 14h. The analysis of exhaled breath from 33 patients and 26 healthy controls revealed that the average accuracy of the training set was 0.81 ± 0.04, with a sensitivity of 0.79 ± 0.08 and a specificity of 0.83 ± 0.00. The utilization of an E-nose can assist doctors in clinical diagnosis and enhance the efficiency of VAP detection. Interstitial lung disease (ILD) is a rare respiratory disease with a global incidence of 0.09% [239]. Due to the overlap of symptoms with other respiratory diseases, community hospitals have a poor ability to identify patients with suspected ILD [240, 241], which affects the timely diagnosis and treatment of patients. Van der Sar et al. [242] evaluated the accuracy of E-nose in distinguishing respiratory characteristics between ILD patients and patients with asthma, COPD, and lung cancer. Using PLS discriminant analysis, the AUC of the test set is 0.99, which has a high potential for identifying ILD, and is helpful for early diagnosis of ILD and distinguishing suspected ILD patients. Wijbenga et al. [243] found that electronic nose technology and pattern recognition methods have good potential in the diagnosis and phenotypic analysis of asthma, COPD and ILD, but have almost no application in the field of lung transplantation. To this end, they studied the diagnostic value of E-nose technology in chronic lung allograft dysfunction (CLAD), CLAD phenotype and CLAD staging of learning to rank. The supervised machine learning method was evaluated on the random training set and the test set, where the AUC of the test set was 0.82. At the same time, they used some known risk factors including age, gender, transplantation type and time as data features to improve the discriminant ability of the model to 0.94.

#### Metabolic Diseases

Metabolic diseases refer to a class of diseases related to the abnormal metabolic function of the body, involving the body's utilization of nutrients, energy production, hormone regulation, and other aspects. For example, diabetes is a chronic metabolic disease associated with defects in insulin secretion or function. The liver plays an important metabolic function in the body, including fat metabolism, glucose metabolism, protein metabolism, etc. Hepatitis, cirrhosis, fatty liver, and other diseases are related to the abnormal structure and function of the liver. Gastrointestinal diseases are usually more related to the structural and functional abnormalities of digestive organs. Metabolic abnormalities may cause a variety of health problems. This section mainly introduces the prediction of diagnosis of gastrointestinal diseases, liver disease, and diabetes based on MOS and related pattern recognition algorithms.

**Fig. 15:**
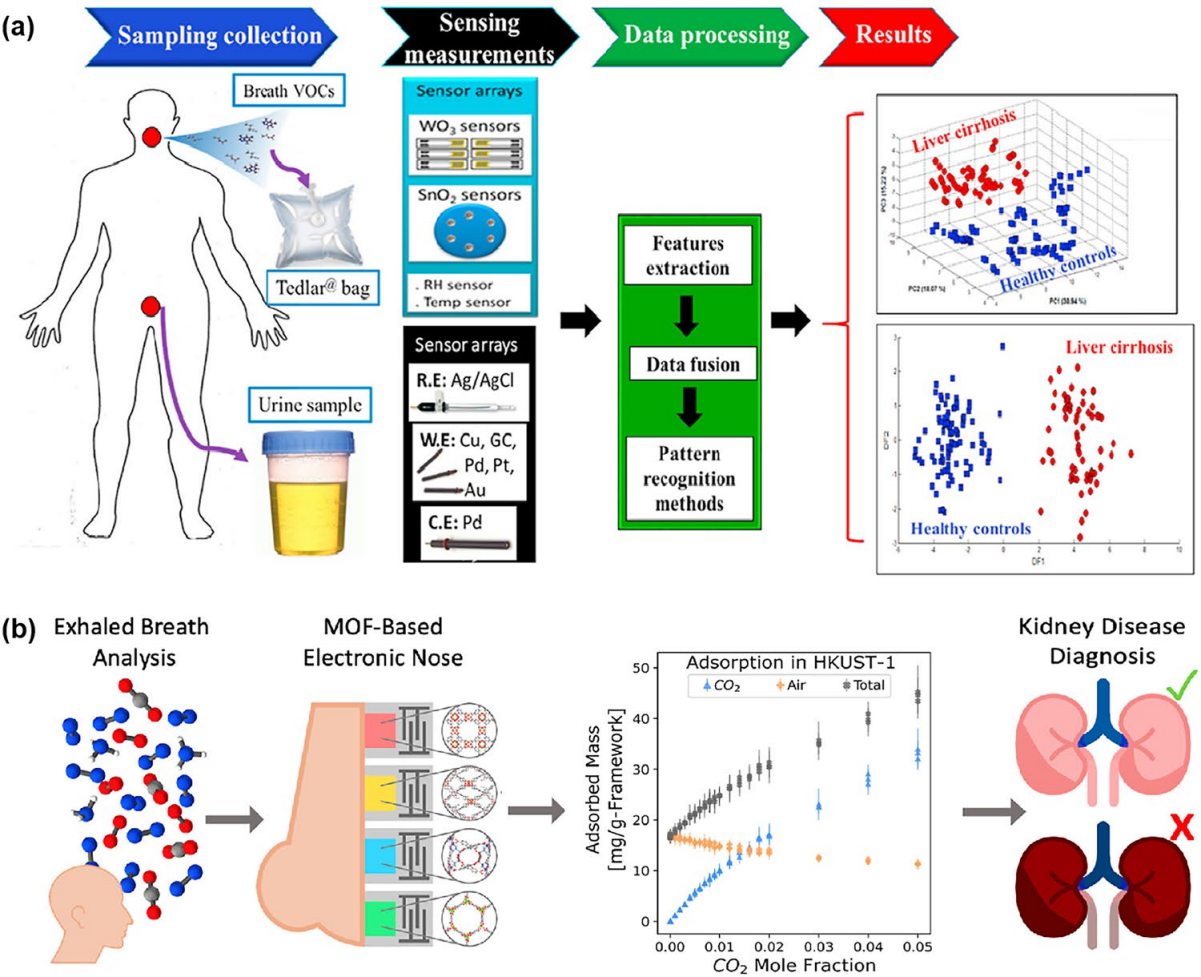
**a** Comparative analysis of the use of E-nose and voltammetric E-tongue for the identification of VOCs in respiratory and urine samples from patients with cirrhosis and healthy controls. Reproduced with the permission from Ref. [248], Copyright Elsevier 2021. **b** MOF-based E-nose combined with CLAC for the detection of kidney diseases. Reproduced with the permission from Ref. [254], Copyright American Chemical Society 2021

The liver, responsible for metabolism and protein absorption, is the main organ of the human body. Liver patients experience metabolic changes due to liver deterioration. Previous studies have demonstrated that isoprene [244], dimethyl sulfide (DMS) [245, 246], and limonene [247] can serve as respiratory markers of cirrhosis. These changes can be detected through respiratory analysis, enabling non-invasive assessment of liver function. In this context, Zaim et al. [248] combined the E-nose and E-tongue. With the aid of pattern recognition methods, they achieved significantly better results in distinguishing liver cirrhosis from healthy individuals using exhaled breath and urine samples compared to using a single system, as depicted in Fig. 15a.

NH_3_ can serve as a marker for kidney disease, as its concentration in the human body increases significantly when the kidney is damaged. The gastrointestinal tract, as the main part of the human digestive system, plays a crucial role in absorbing nutrients and eliminating waste. In recent studies, various volatile chemical biomarkers have been discovered in the intestinal tract of patients [249]. These biomarkers have shown potential in detecting different disease states within the gastrointestinal tract [250, 251]. For instance, Arasaradnam et al. [252] utilized an E-nose to monitor VOCs present in the urine tract successfully identifying patients with inflammatory bowel disease (IBD). Through PCA and DFA, the E-nose demonstrated its ability to distinguish between 48 IBD patients and 14 healthy controls, as well as differentiate between active and remission diseases. Utilizing pattern recognition methods can further enhance the sensitivity and specificity of the E-nose in diagnosing VOCs. For example, in the diagnosis of infectious colitis, McGurie et al. [253] employed short multi-capillary chromatography column and MOS sensors combined with an ANN to analyze VOCs in the headspace of 100 fecal samples (positive and negative samples accounted for half and half respectively). The results of the ANN analysis of the data indicated that despite the small sample size, the sensitivity and specificity were 85% and 80% respectively, which was comparable to the results of the commercial test. McGuire et al. [253] also utilized the ANN algorithm to examine fecal samples for the detection of Clostridium difficile-infected Stool and achieved a sensitivity of 80% and specificity of 85%. A new numerical algorithm, which combined linear adsorption coefficients, was proposed by Day et al. [254], aiming to quantify the composition of respiratory samples. They used five metal–organic framework (MOF)-based sensors to test 100 anhydrous respiratory kidney disease samples by this method, and successfully quantified the nitrogen content in all samples, as shown in Fig. 15b.

Diabetes is an insulin-related disease and is the most common metabolic disorder. Research has indicated that diabetic patients have a concentration of acetone in their exhaled breath that exceeds 1.7 ppm, which is at least 118 times higher than that of healthy individuals [255]. This disparity in acetone concentration can be utilized for diagnosing diabetes by analyzing the breath. In fact, in 1997, Ping et al. [256] proposed a non-invasive diagnostic method for diabetes using an E-nose. They employed a clustering algorithm to analyze the sensor response data and clustered samples from 18 diabetic patients and 14 normal individuals separately. Furthermore, Jin et al. [257] utilized PCA and Euclidean distance comparison to identify the sensor array with the best selection performance in differentiating acetone from various interfering gases in exhaled breath. They successfully detected five VOCs, including acetone, using real breath samples from 12 diabetic patients and 13 healthy controls. The PCA analysis of the test data illustrated that the five gases could be accurately distinguished. Additionally, apart from analyzing respiratory gases, the gases and vapors emitted from urine can also be examined. Esfahani et al. [258] conducted a study to investigate the feasibility of using urinary VOCs as biomarkers for diagnosing diabetes. The researchers employed FOX 4000 and FAIMS E-nose instruments to analyze volatile compounds in urine samples. They utilized four classifiers, namely sparse logistic regression, RF, Gaussian Process, and SVM, for classification purposes. The findings from both E-noses demonstrated that the experimental urine VOCs can effectively distinguish diabetes mellitus type 2 (DM2) from the healthy control group. Additionally, the authors suggest that the accuracy of the E-nose may be influenced by the storage time of the urine samples. Similarly, Lee et al. [259] used four CuO-based gas sensor arrays to detect VOCs related to exhaled breath and employed PCA to differentiate acetone gas from other VOCs gases such as ethanol and formaldehyde. The sensor array displayed a response to acetone as low as 9 ppb, indicating its potential for early detection of diabetes. The PCA score plot demonstrated a clear separation between acetone and the other tested VOCs (ethanol and formaldehyde).

## Summary and Prospect

Aiming to overcome the cross-sensitivity in chemiresistive gas sensors, this review comprehensively discusses a variety of pattern recognition methods based on the sensor array. The review delves into the advantages and disadvantages of different algorithms within the realm of gas recognition and their respective application scenarios. Traditional machine learning methods are well-suited for scenarios with limited resources or where rapid deployment is essential due to their modest data requirements, strong interpretability, and low computational complexity. These methods are particularly useful in situations like chemical monitoring, where the interpretability of models is crucial. On the other hand, neural network algorithms such as FNN, CNN, and RNN excel in processing intricate data relationships and patterns by automatically extracting features, making them more appropriate for recognizing multi-component gas mixtures within extensive datasets. When faced with gas identification challenges characterized by high complexity and data interference, ensemble learning methods enhance prediction accuracy and robustness through model fusion. In practical applications, it is often necessary to compare various methods to identify the most suitable strategy for efficiently and accurately completing gas identification tasks. Furthermore, with the ever-increasing development trend of IoT, the advancement of multi-sensor array cloud-edge-end integration is poised to revolutionize applications across diverse industries such as food safety, environmental protection, and disease diagnosis.

## Challenges

As the proverb says, every coin has two sides. The cross-sensitivity of gas sensors can weaken the accurate recognition of target gases in complex environments and limit their application range. However, this challenge presents an opportunity to identify multiple gas species and concentrations using pattern recognition methods. Gas identification difficulty often stems from the complexity of mixed compounds in the air, as seen in the more than 3,000 types of exhaled gases discovered. Utilizing pattern recognition, it is possible to accurately detect multiple gases with a small number of sensors in specific environments, which shows great promise. Despite the progress made, the following challenges may arise when applying pattern recognition technology to artificial olfactory systems:

1. Sensor drift compensation: Machine learning uses historical and real-time data to predict sensor behavior. Compared to hardware-based solutions, implementing machine learning for drift compensation is cost-effective, extends sensor lifetimes, and offers flexibility. However, machine learning models face challenges in data collection, model updating, generalization, online learning, and adaptability. Managing model complexity, computational demands, interpretability, and long-term performance are also critical. Addressing these requires strategies like incremental learning, adaptive algorithms, and ensemble modeling.
2. Scalable models with dimensionality fusion: The complexity of handling high-dimensional data and integrating multiple sensors poses a challenge in the context of multi-sensor fusion. Exploring efficient techniques for reducing data dimensionality and seamlessly integrating multimodal sensor data is crucial to simplifying input complexity. Additionally, scalable and transferable machine learning models are needed to adapt easily across diverse E-nose platforms and application domains, thereby reducing the reliance on extensive domain-specific training data.
3. Data interpolation issue: Interpolation techniques are common in processing VOC sensor data, while simple interpolation methods may fail to capture complex nonlinear relationships, resulting in generating data that may lack real-world significance. Machine learning methods can enhance data generation techniques like generative learning to produce samples closer to real data. Future exploration should focus on adaptive algorithms with continuous learning and feedback mechanisms to adapt to dynamic data changes.
4. Real-time monitoring: In the context of real-time monitoring, rapid data processing and response are essential. Models need to continuously learn and update from new data to adapt to dynamic changes in data and sensor drift. Traditional machine learning models face challenges in effectively handling rapidly changing data patterns or real-time events. Deep learning models for electronic noses can circumvent manual feature extraction, accepting streaming data acquired as time windows, while leveraging cloud computing platforms to accelerate real-time prediction and analysis.

## Prospects

The rapid development of IoT has brought unprecedented convenience to people’s lives, bringing a growing market for mobile devices, and E-noses will also face new opportunities. For the cross-sensitivity of gas sensors, the development of pattern recognition methods will also usher in new directions:

1. Integration with IoT and edge computing: The integration of E-nose with the IoT and edge computing presents a promising avenue for real-time monitoring and analysis. Machine learning algorithms optimized for low-power, edge devices can enable decentralized, immediate decision-making.
2. Advanced deep learning techniques: Leveraging recent advancements in deep learning, such as transformer models, reinforcement learning, and online learning, can significantly enhance the analytical capabilities of E-noses, enabling them to learn from limited data and generalize across diverse conditions.
3. Sense-storage-computing integrate: The development of an integrated olfactory chip that combines sensing, data storage, and computational capabilities represents a significant leap forward. Utilizing memristor arrays, these chips could mimic the human olfactory system’s efficiency and adaptability, leading to compact, energy-efficient E-noses with enhanced sensitivity and selectivity.
4. Large model calculations of molecular: Bridging the gap between theoretical molecular and chemical models and empirical sensor data can greatly enhance understanding and prediction of sensor behavior in complex environments. By integrating large-scale models of molecular interactions with real-world sensor outputs, researchers can develop more robust and accurate predictive models for odor detection.

In conclusion, the continuous innovation of chemiresistive gas sensor technology and the development of pattern recognition methods are expected to extend the application of intelligent olfactory systems to other scenes. Overcoming current challenges not only improves the selectivity of chemical gas sensors but also unleashes their full potential to meet the critical needs of various industries.
